# Brain functional connectivity predicts depression and anxiety during childhood and adolescence: A connectome-based predictive modeling approach

**DOI:** 10.1162/IMAG.a.145

**Published:** 2025-09-12

**Authors:** Francesca Morfini, Aaron Kucyi, Jiahe Zhang, Clemens C.C. Bauer, Paul A. Bloom, David Pagliaccio, Nicholas A. Hubbard, Isabelle M. Rosso, Anastasia Yendiki, Satrajit S. Ghosh, Diego A. Pizzagalli, John D.E. Gabrieli, Susan Whitfield-Gabrieli, Randy P. Auerbach

**Affiliations:** Department of Psychology, Northeastern University, Boston, MA, United States; Department of Psychological and Brain Sciences, Drexel University, Philadelphia, PA, United States; Center for Precision Psychiatry, Massachusetts General Hospital, Boston, MA, United States; Department of Brain and Cognitive Sciences and McGovern Institute for Brain Research, Massachusetts Institute of Technology, Cambridge, MA, United States; Department of Psychiatry, Columbia University, New York, NY, United States; Division of Child and Adolescent Psychiatry, New York State Psychiatric Institute, Columbia University, New York, NY, United States; Department of Psychology, University of Nebraska-Lincoln, Lincoln, NE, United States; Center for Depression, Anxiety, and Stress Research, McLean Hospital, Belmont, MA, United States; Department of Psychiatry, Harvard Medical School, Boston, MA, United States; Athinoula A. Martinos Center for Biomedical Imaging, Massachusetts General Hospital, Charlestown, MA, United States

**Keywords:** depression, anxiety, adolescence, functional connectivity, functional magnetic resonance imaging, longitudinal studies, machine learning

## Abstract

Identifying brain-based correlates of risk for future depression and anxiety severity in youth could improve prevention and treatment efforts. We tested whether connectome-based predictive modeling (CPM) based on resting-state functional connectivity (FC) at baseline: (a) predicts future depression and anxiety severity during childhood and (b) generalizes to adolescence. We used two independent, longitudinal datasets including children from the Adolescent Brain Cognitive Development (ABCD) study and adolescents from the Boston Adolescent Neuroimaging of Depression and Anxiety (BANDA). ABCD included a cohort of 11,875 children ages 9–11 years old, and BANDA enrolled 215 adolescents ages 14–17 years, of which ~70% reported a depressive or anxiety disorder. CPM with internal (within ABCD) and external validation (from ABCD to BANDA) used baseline whole-brain FC to predict depression and anxiety severity at a 1-year follow-up assessment. ABCD-derived functional connections, which we term “Symptoms Network”, were validated within BANDA to test model applicability in adolescence, which is a peak period for the emergence of internalizing disorders. Participants with complete data were included from ABCD (n = 3,718, 52.9% girls, ages 10.0 ± 0.6) and BANDA (n = 150, 61.3% girls, ages 15.4 ± 0.9). In ABCD, we found that FC predicted 1-year follow-up symptoms severity (*ρ* = 0.058, *p* = 0.040), measured with the Child Behavior Checklist Anxious/Depressed subscale. External validation in BANDA indicated that the Symptoms Network predicted 1-year follow-up symptoms severity (*ρ* = 0.222, *p* = 0.007), measured with the Revised Child Depression and Anxiety Scale *t*-transformed total score. In both ABCD and BANDA, FC enhanced the prediction of future symptom severity beyond baseline clinical and demographic information (baseline severity, sex, and age), including when correcting for mean head motion. The ABCD-derived connections included contributions from somatomotor, attentional, and subcortical regions and were characterized by heterogeneous FC within adolescents, where the same region pairs were characterized by positive FC for some participants but by negative FC for others. In conclusion, FC may provide inroads for early identification of internalizing symptoms, which could inform preventative-intervention approaches prior to the emergence of affective disorders during a critical period of neuromaturation. However, the small effect sizes and heterogeneity in results underscore the challenges of employing brain-based biomarkers for clinical applications and emphasize the need for individualized approaches for understanding neurodevelopment and mental health.

## 1 Introduction

Internalizing disorders, such as depression and anxiety, often co-occur throughout the course of childhood and adolescence (Auerbach et al., 2022) and are common and debilitating (Avenevoli et al., 2015; Essau, 2003, 2008; Kessler et al., 2005). Early onset of either anxiety or depression contributes to a more persistent (Batelaan et al., 2014; Beesdo et al., 2007; Kessler et al., 2012) and more complex psychiatric comorbidity during adulthood (Auerbach et al., 2016, 2018). Several initiatives—such as the Human Connectome Project (HCP) and the Adolescent Brain Cognitive Development (ABCD) study—provide large-scale and specialized neuroimaging datasets that can characterize neurodevelopmental vulnerability to internalizing disorders. These datasets, combined with recent advances in computational approaches, have enormous potential to augment our understanding of brain mechanisms related to depression and anxiety, which may improve our understanding of mental health as it develops in youth.

Functional connectivity (FC) during a resting state has identified promising neural correlates of depression and anxiety in functional magnetic resonance imaging (fMRI) studies. Depression is often characterized by altered FC, including hyperconnectivity of the default mode network (DMN), hyperconnectivity between the DMN and the frontoparietal network (FPN) often referred to as the central executive network (but see Uddin et al., 2023 for a discussion about network nomenclatures), and hypoconnectivity between FPN and dorsal attention network (DAN; Kaiser et al., 2015). With respect to anxiety disorders, research has observed within-network hypoconnectivity of the DMN, FPN, and salience network (or ventral attention network; VAN), DMN-FPN hypoconnectivity, and VAN-sensorimotor network (SMN) hypoconnectivity (Xu et al., 2019). However, these findings often rely on cross-sectional studies of adults (MacNamara et al., 2016) with fewer studies focusing on longitudinal characterization during development.

The availability of open-access, longitudinal datasets coupled with advancements in computational approaches (e.g., machine learning-based predictive models) has propelled research focused on FC linked to internalizing symptoms in youth (Gracia-Tabuenca et al., 2024; Toenders et al., 2019). Studies suggest that distributed connectivity patterns among frontal, parietal, and subcortical regions predict symptom severity in healthy or depressed-anxious children (Ho et al., 2022; Whitfield-Gabrieli et al., 2020), female adolescents with no history of depressive disorder (Jin et al., 2020), adolescents with internalizing disorders (Chahal, Kirshenbaum, et al., 2021; Chahal, Weissman, et al., 2021), and unaffected college students (He et al., 2021). Yet, to date, no study has investigated FC as a predictor of prospective symptom severity in children and then examined the generalizability to adolescents in independent data. This data-driven approach would enable the detection of neural patterns in children and extend them to adolescents, establishing whether mechanisms associated with mental health and neural development in childhood are also core to adolescence.

To address this gap, we identified FC predictors of internalizing symptoms over 1 year in predominantly healthy children and then tested whether these patterns predicted prospective anxious and depressive symptom severity in an independent sample of predominantly clinical (~70%) adolescents. Connectome-based predictive modeling (CPM; Shen et al., 2017), a data-driven machine-learning approach designed to investigate brain associations with continuous phenotypes, was applied in two independent, longitudinal, and clinically-heterogeneous datasets. The discovery dataset comprised a large community cohort from the ABCD study (Barch et al., 2018) of predominantly healthy children recruited during a peak time period for the emergence of anxiety disorders. The extension dataset included adolescents from the Boston Adolescent Neuroimaging of Depression and Anxiety (BANDA; Hubbard et al., 2020, 2024) HCP, a study that aimed to investigate depression and anxiety during a period of heightened vulnerability for internalizing disorders. Based on prior fMRI-FC studies investigating longitudinal predictors of depression and anxiety in youth (Ho et al., 2022; Whitfield-Gabrieli et al., 2020, see also reviews; Macêdo et al., 2022; Toenders et al., 2019; van Tol et al., 2021), we expected that distributed FC patterns, including subcortical, occipital, and frontal regions, would predict internalizing symptoms across samples.

## 2 Methods

### 2.1 Overview of datasets

**Fig. 1.**
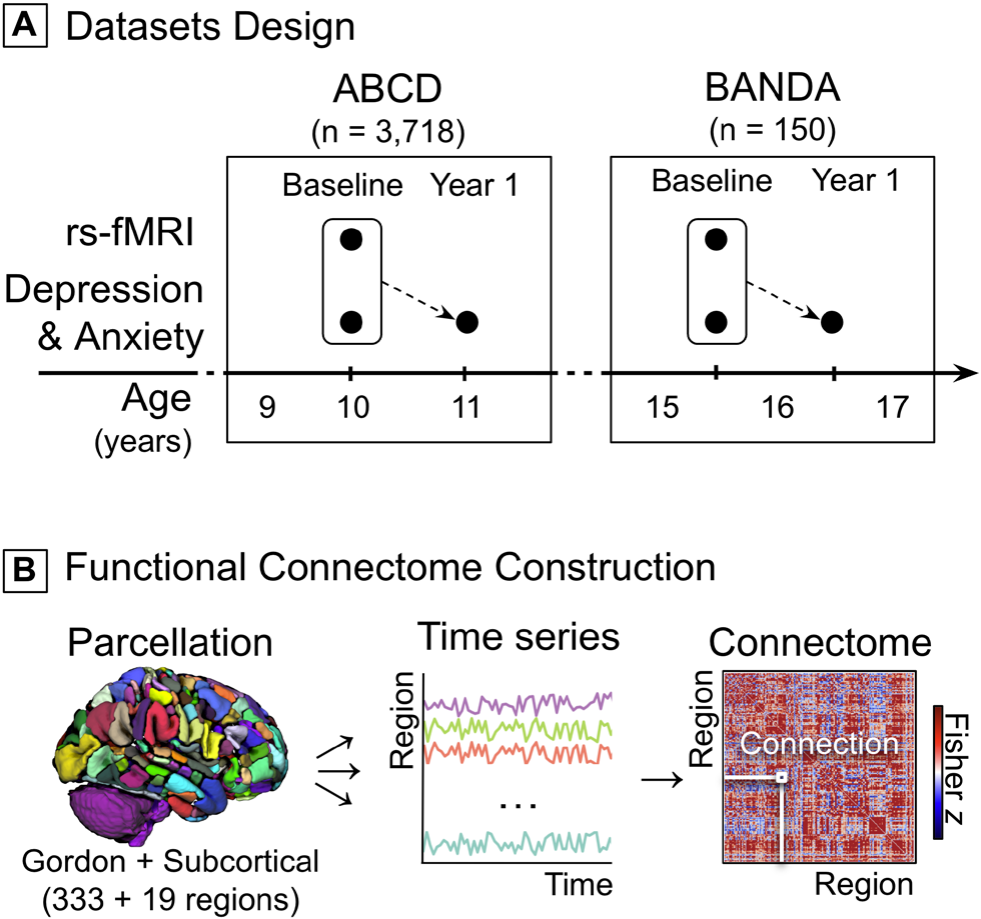
Dataset design and connectomes construction. (A) Schematic representation of ABCD and BANDA study designs as a function of mean participant age at each study visit. Black dots indicate when rs-fMRI data and symptom severity reports were acquired. (B) rs-fMRI data for each participant were parcellated into 333 cortical (Gordon et al., 2016) and 19 subcortical (Fischl et al., 2002). The time series of each region was correlated with that of every other region to form a participant-specific connectome, wherein each region-to-region correlation represents a functional connection. Correlation coefficients were Fisher-z transformed. ABCD: Adolescent Brain Cognitive Development Study; BANDA: Boston Adolescent Neuroimaging of Depression and Anxiety; rs-fMRI: resting-state functional magnetic resonance imaging.

Two independent longitudinal datasets were included (Fig. 1A). ABCD was utilized as the discovery dataset in CPM, and BANDA was leveraged to test the generalizability (for participant information, study design, symptom assessment, MRI data and MRI processing for ABCD, see Supplementary Appendices S1 and S2, and for BANDA Supplementary Appendices S3 and S4). For both datasets, each site received Institutional Review Board approval from their institution and written informed consent and assent were received by the guardian and participating children.

#### 2.1.1 ABCD: Discovery dataset

ABCD is a publicly available, longitudinal, multi-site study in children (N = 11,875 from 21 sites) which recruited children from the community and assessed the presence of mental disorders but did not specifically recruit clinical populations. At baseline, participants were ages 9–11 years. Demographic and clinical information were obtained from the Annual Curated ABCD 4.0 Data Release (Barch et al., 2018). Resting-state fMRI (rs-fMRI) data (~20 minutes across four runs) were obtained from the ABCD-BIDS Community Collection 3165 (Feczko et al., 2021) and only included MRI data that passed the Data Analysis Imaging Center quality control (Chai et al., 2012). These data had been fully preprocessed and analyzed by the DCAN Lab via the ABCD-BIDS MRI pipeline (Feczko et al., 2021), which had been adapted from the HCP minimal preprocessing pipeline (Glasser et al., 2013). The child’s depression and anxiety symptom severity was indicated via the Child Behavior Checklist (CBCL; Achenbach, 1991) Anxious/Depressed subscale *t*-transformed scores. The Anxious/Depressed subscale measures symptoms of anxiety and depression in children, such as excessive worrying, sadness, withdrawal, and nervousness, based on caregiver reports. It includes 13 items (range [0, 26]) with higher scores indicating greater symptom severity. See Supplementary Appendix S1 for further details.

We analyzed data collected between September 2016 and March 2020, which reflected the baseline and 1-year follow-up (Fig. 1A). The final sample included children (n = 3,718; Supplementary Fig. S1A) with available symptom severity reported at the baseline (CBCL_base_) and 1-year follow-up (CBCL_y1_), with no other family member scanned at another MRI site, and with at least 10 minutes of low-motion baseline rs-fMRI (FD < 0.25 mm).

#### 2.1.2 BANDA: Extension dataset

BANDA is a publicly available, longitudinal, single-site dataset that investigated depression and anxiety in adolescents (Hubbard et al., 2020, 2024; Siless et al., 2020). Our research team collected these data from October 2016 to November 2021 and enrolled depressed-anxious and healthy adolescents (N = 215) ages 14–17 years. Data were obtained from the BANDA 1.1 Data Release (Hubbard et al., 2024). MRI (~23 minutes across four runs) data were pre-processed via the HCP minimal preprocessing pipeline (Glasser et al., 2013) and underwent fMRI quality control procedures (Morfini et al., 2023). Symptom severity was assessed via the self-report Revised Child Depression and Anxiety Scale (RCADS) *t*-transformed total scores (de Ross et al., 2002) which assess overall levels of anxiety and depressive symptoms in youth, covering multiple specific disorders (e.g., generalized anxiety, social phobia, panic disorder, major depression) through self-report. See Supplementary Appendix S2 for further details. The final sample included adolescents (n = 150; Fig. 1A) with available symptom severity reported at the baseline (RCADS_base_) and 1-year follow-up (RCADS_y1_), and with low-motion rs-fMRI data at baseline (Supplementary Fig. S1B).

### 2.2 Functional connectomes construction

The degree to which different regions of the brain have synchronized activity can be summarized in a functional connectome, that is, a matrix of the correlations between the time series of every brain region to that of every other region. Connectomes for ABCD rs-fMRI data were constructed and released by the DCAN lab, with the brain parcellated into 352 regions (333 cortical from Gordon et al., 2016, and 19 subcortical from Fischl et al., 2002). For consistency, we adopted the same parcellation and method to build the connectomes for the BANDA adolescents (Fig. 1B), by calculating Fisher r-to-z-transformed Pearson’s correlation coefficients between the time series of every region-to-region pair (i.e., one functional connection). This resulted in 61,776 unique functional connections, that is, [352 × (352-1)] / 2 unique connections, which were used as predictors in CPM.

### 2.3 ABCD Connectome-based predictive modeling analyses

**Fig. 2.**
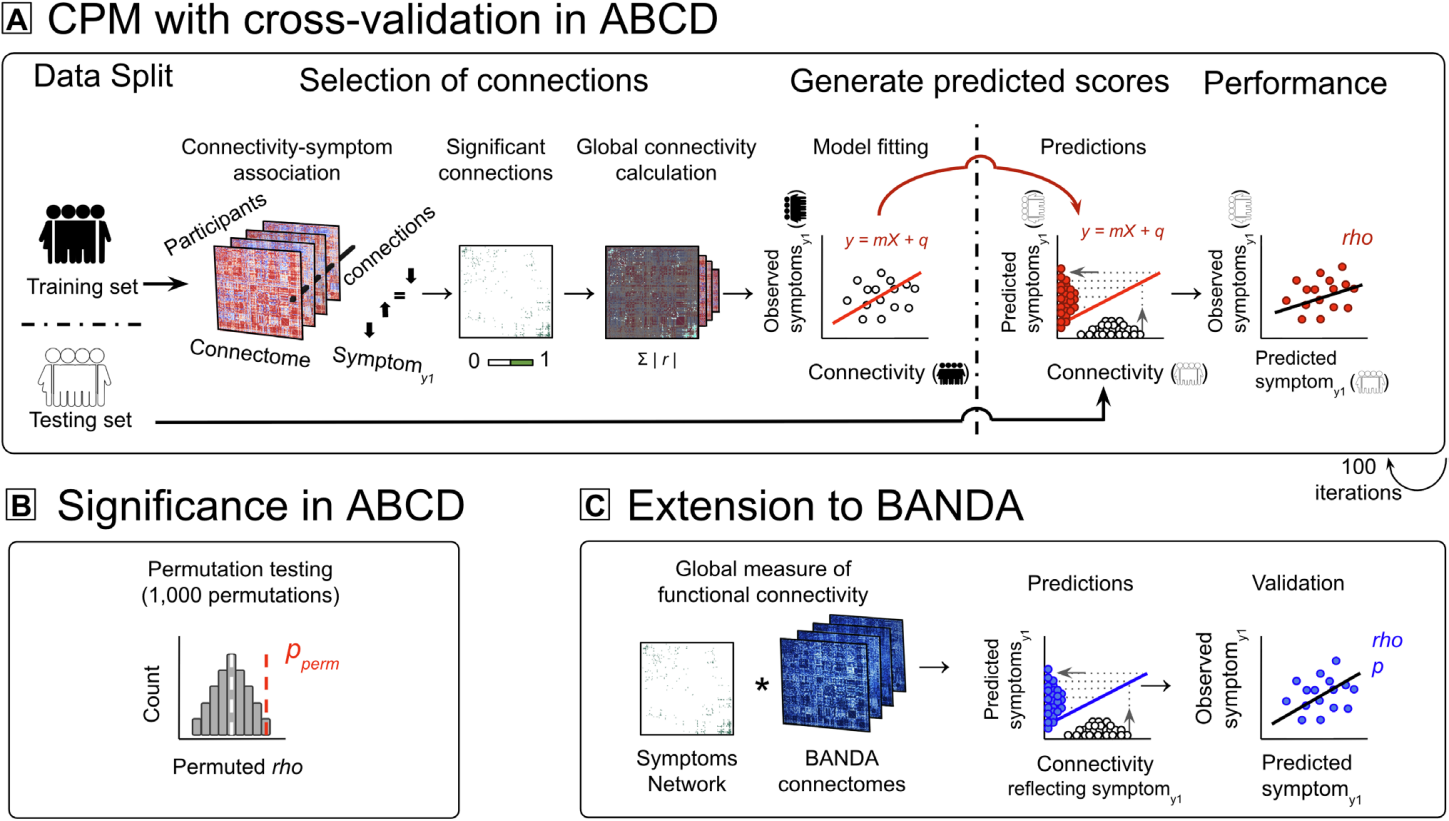
Connectome-based predictive modeling in ABCD and external validation in BANDA. (A) In ABCD participants, CPM with 100 iterations and 1,000 permutations of leave-half-sites-out cross-validation used baseline whole-brain FC to predict symptom severity_y1_, correcting for symptom severity_base_, sex at birth, age, and mean head motion. The ABCD dataset was evenly split into a training and testing set. Within the training set, functional connections significantly correlated with symptom severity_y1_ at *p* < 0.001 were retained. Network strengths were calculated as the sum of connection weights (i.e., absolute *z*-values). Network strengths and symptom severity_y1_ were fit with a linear model in the testing set. The fitted line was used to generate predicted symptom severity_y1_ scores from FC in the testing set. Networks’ prediction performance was evaluated as the mean correlation between the observed and predicted symptom severity_y1_ scores. (B) Network’s prediction significance was assessed via nonparametric permutation testing with 1,000 permutations using randomly shuffled data. Significance (*p_perm_*) was defined as the percentage of permuted *ρ* values larger than the *ρ* value generated from the observed data. (C) The ABCD-derived Network was externally validated in the independent BANDA dataset. Network strength scores were calculated for each BANDA adolescent and used to predict symptom severity_y1_. Generalizability was defined as the Spearman’s correlation between the network strengths and symptom severity_y1_, correcting for symptom severity_base_, sex at birth, age, and mean head motion. ABCD: Adolescent Brain Cognitive Development Study: BANDA, Boston Adolescent Neuroimaging of Depression and Anxiety; CPM: Connectome-Based Predictive Modeling; y1: 1-year follow-up assessment; FC: Functional Connectivity.

CPM with leave-half-sites-out cross-validation (recommended for large sample sizes; Scheinost et al., 2019) consisted of five broad steps (Fig. 2A, B). *First*, all ABCD children acquired from a random selection of half of the ABCD sites were assigned to either a training or a testing set (Fig. 2A) and kept separate for all procedures of the same iteration. In light of the multi-site and nested nature of ABCD (which comprises members of the same family), splitting participants based on MRI site minimized the risk of information leakage between sets (Rosenblatt et al., 2024) and allowed to test for generalizability between different MRI sites within the ABCD dataset. *Second*, within the training set only, functional connections that correlated with CBCL_y1_ (i.e., depression and anxiety severity at the 1-year follow-up) at a Spearman’s rank correlation *p* < 0.001 were retained (Shen et al., 2017). We used Spearman’s, rather than Pearson’s, correlation to minimize the effect of outliers given that CBCL scores were positively skewed (Supplementary Fig. S2). *Third*, for each child, we calculated a composite FC measure (i.e., network strengths; Shen et al., 2017) as the sum of the *z* absolute values of the connections comprising the Network. Network strengths and CBCL_y1_ in the training set were fit with a linear regression model. The fitted line was then used to generate CBCL_y1_ predicted scores from network strengths in the testing set. Note that in the testing set pipeline, the CBCL_y1_ observed scores were not used and the CBCL_y1_ predicted scores were derived purely from network strengths (i.e., FC). *Fourth*, the networks’ prediction performance was assessed in the testing set by computing a Spearman’s partial correlation to compare observed versus predicted CBCL_y1_, while correcting for symptom severity at baseline (i.e., CBCL_base_), sex at birth, age, and mean head motion. These four steps were iterated 100 times generating a set of Spearman’s *ρ* values which were averaged to represent the ability of the Network to predict CBCL_y1_ within ABCD children. *Fifth*, as the training and testing sets were not independent across iterations resulting in an overestimation of the degrees of freedom in parametric statistics, the significance of the Network’ prediction was assessed via nonparametric permutation testing (Fig. 2B). That is, the above four steps were permuted 1,000 times using randomly shuffled data (i.e., the connectome of a participant was used to predict the CBCL_y1_ of another random participant) generating a set of plausible, yet not-observed, connectivity-to-symptom matches. The permuted *ρ* values generated represented an empirical null distribution against which we compared the prediction generated by the observed data. Specifically, the Network’ prediction significance (*p_perm_*) was defined as the proportion of permuted *ρ* values larger than the true *ρ* values (Shen et al., 2017). That is, the Network was considered to be significantly predictive only if less than 5% of the 1,000 predictions generated from shuffled data outperformed the prediction generated from observed data. This approach guards against the potential bias of using correlation in well-powered samples as it focuses on whether the observed effect is unusual, rather than simply significant, as compared to other scenarios which would have had a similar likelihood of being driven by sample size alone.

### 2.4 BANDA extension analyses

To test the generalizability of the predictions from the ABCD children to the prediction of prospective symptom severity in adolescents, we externally validated within BANDA (Shen et al., 2017) the ABCD-derived set of connections (Fig. 2C), which we refer to the “Symptoms Network” for brevity (but see Uddin et al., 2023 for some controversies regarding network nomenclature). The Symptoms Network, generated from all ABCD children, were used to generate composite measures of FC (i.e., network strengths) from the connectomes of each BANDA adolescent. These composite FC measures generated in BANDA were correlated with RCADS_y1_, using Spearman’s partial correlation (correcting for RCADS_base_, sex at birth, age, and mean head motion), effectively testing whether FC predicted prospective depression and anxiety symptom severity in an independent cohort of adolescents mostly affected by depression and anxiety.

## 3 Results

### 3.1 Participants

**Fig. 3.**
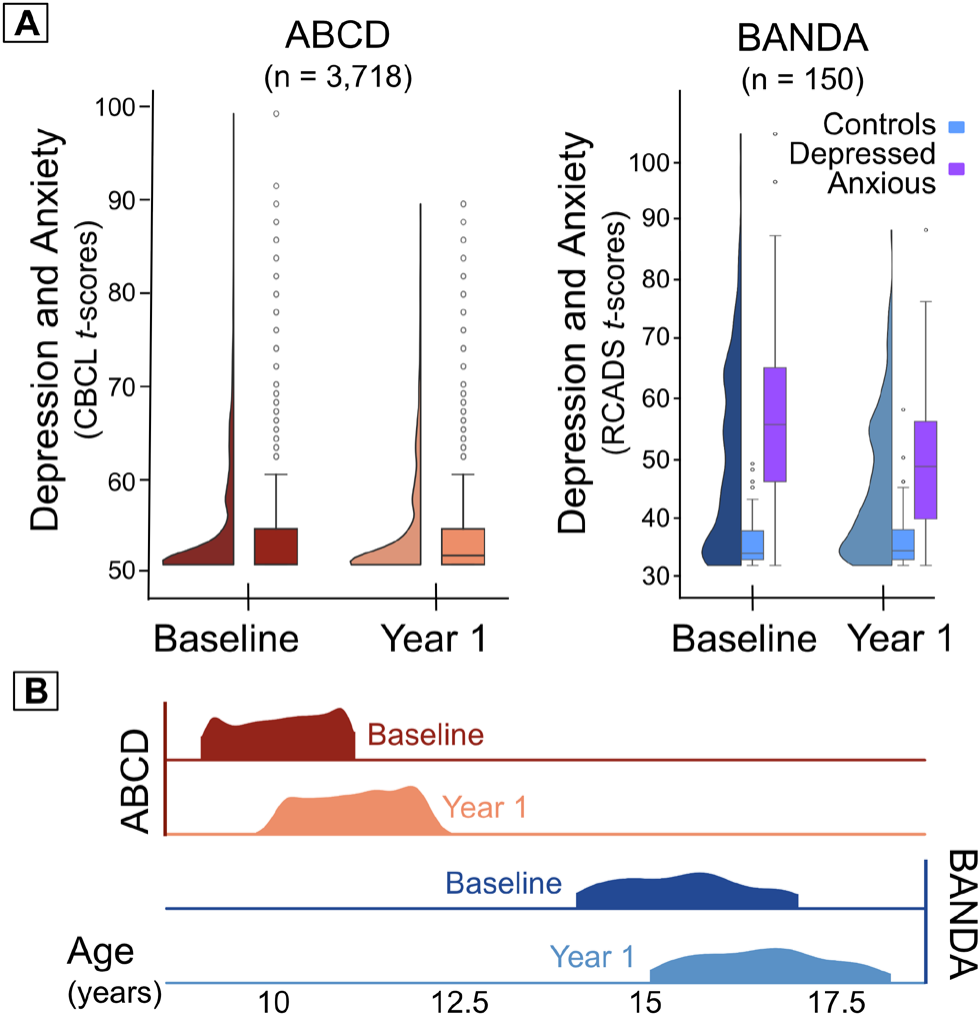
Depression and anxiety severity and age distributions in ABCD children and BANDA adolescents. (A) Distributions of depression and anxiety severity scores for ABCD (left; CBCL_base_ = 53.48 ± 6.06; CBCL_y1_ = 53.51 ± 6.02) and for BANDA (right; RCADS_y1_ = 49.11 ± 15.28; RCADS_base_ = 44.62 ± 11.39). Controls and Depressed/Anxious group assignments in BANDA were based on a clinician evaluation of diagnoses as per the Diagnostic and Statistical Manual of Mental Disorders 5th edition (American Psychiatric Association, 2013) assessed with the Kiddie Schedule for Affective Disorders and Schizophrenia Present and Lifetime Version (Kaufman et al., 1997). (B) Age distributions at the time of study assessments in ABCD children (top, red distributions) and BANDA adolescents (bottom, blue distributions). ABCD: Adolescent Brain Cognitive Development Study; BANDA: Boston Adolescent Neuroimaging of Depression and Anxiety; CBCL: Child Behavior Checklist, Anxious/Depressed subscale *t*-transformed scores; RCADS: Revised Child Depression and Anxiety Scale, *t*-transformed total scores.

Demographic and clinical characteristics of the included ABCD children and BANDA adolescents are summarized in Figure 3 and Supplementary Tables S1–S3. Depression and anxiety severity—on average—remained stable in ABCD (CBCL_base_ = 53.51 ± 6.02; CBCL_y1_ = 53.48 ± 6.06) and decreased, that is, improved, in BANDA (RCADS_base_ = 49.11 ± 15.28; RCADS_y1_ = 44.62 ± 11.39) mostly driven by the depressed-anxious participants (RCADS_base_ = 56.47 ± 14.26; RCADS_y1_ = 49.07 ± 11.50) while the healthy group reported stable symptom severity between study visits (RCADS_base_ = 36.04 ± 4.43; RCADS_y1_ = 36.70 ± 5.25). Symptom severity scores reported at the baseline and at the 1-year follow-up assessments were correlated (Supplementary Fig. S2) in both ABCD children (*r* = 0.68, *p* = 0.001) and BANDA adolescents (*r* = 0.63, *p* = 0.001). Self-reported depression and anxiety severity (RCADS subscales) were strongly correlated in adolescents both at baseline (Pearson’s two-sided *r* = 0.77 ± 0.11, *p-corrected _Bonferroni_* < 0.0001, *r* range [0.56, 0.99]) and at the 1-year follow-up ((*r* = 0.73 ± 0.14, *p-corrected _Bonferroni_* < 0.0001, *r* range [0.42, 0.99]); Supplementary Table S4).

### 3.2 ABCD connectome-based predictive modeling

CPM revealed that baseline FC significantly predicted CBCL_y1_ (correcting for CBCL_base_, sex at birth, age, and mean head motion) in ABCD children (*ρ* = 0.058, *p_perm_* = 0.040) reliably across multiple iterations of cross-validation and permutation testing. As expected, the distribution of shuffled-predicted correlations (Supplementary Fig. S3) was centered around *ρ* = 0 but could range between negative and positive values. A negative correlation in this context (i.e., between predicted and observed scores) would have represented cases where the predictions were inaccurate.

Sensitivity analyses suggest that these effects were robust to employing varied CPM analytical parameters, including different *p*-value thresholds for connection selection (0.05, 0.01, or 0.005) and for using raw rather than *t*-transformed severity scores severity scores (*ρ* = 0.062 ± 0.003 with range [0.058, 0.065]; *p* = 0.029 ± 0.011 with range [0.012, 0.038]). Additionally, there were no significant associations between in-scanner mean head motion (Supplementary Fig. S2), a confounding factor that artificially increases prediction performance if correlated with the predicted variable (Shen et al., 2017), and CBCL_base_ (*r* = 0.01, *p* = 0.703) or CBCL_y1_ (*r* = -0.02, *p* = 0.177).

These results were generated based on iterations of ABCD subsets of participants via the cross-validation approach and showed that results were reliable and robust regardless of the specific subset of individuals considered. Accordingly, we described and tested the performance of the connections that significantly correlate with CBCL_y1_ scores (adjusted for CBCL_base_, sex, age, and mean head motion) in all ABCD children. For brevity, we refer to this set of brain functional connections as the “Symptoms Network”, which reflects results from the model: depression and anxiety *t*-transformed scores _y1_ = depression and anxiety *t*-transformed scores _base_ + sex _base_ + age _base_ + mean head motion _base_ + functional connectivity _base_.

### 3.3 Contextualizing the symptoms network

**Fig. 4.**
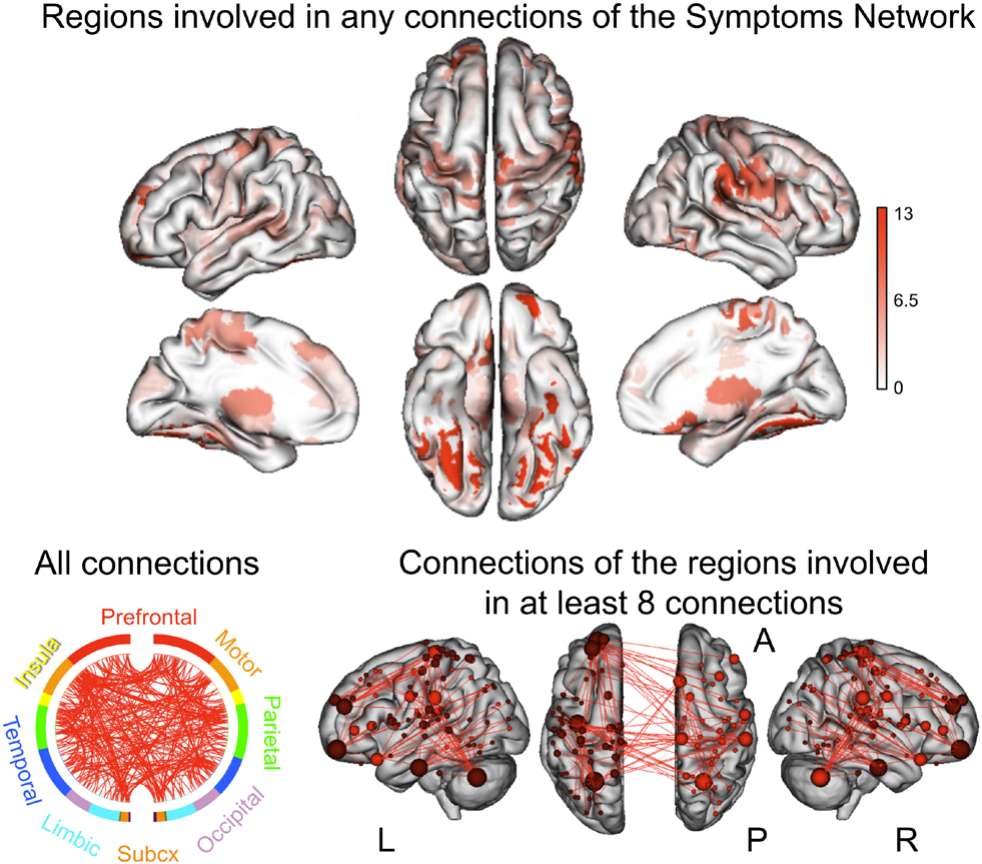
Symptoms Network. CPM identified a predictive network in ABCD representing functional connections correlated with CBCL_y1_ correcting for CBCL_base_, sex, age, and mean head motion. The top panel reflects the distribution of the regions involved in at least one connection with another region in the Symptoms Network, color coded by absolute connection count. The chord plot (bottom left) depicts the spatial distribution of all identified connections based on anatomical macroscale lobe definition. The brain images (bottom right) depict the spatial distribution and degree (i.e., number of non-zero connections represented by the size of the node) of the Symptoms Network. For visualization purposes, only regions with degree ≥ 8 are displayed on the brain rendering images.

The Symptoms Network was characterized by connections distributed across the brain (Fig. 4), comprising 251 unique connections representing 0.41% of all possible connections.

To aid results interpretability, we grouped each cortical region to one of seven canonical brain networks (Yeo et al., 2011; see Supplementary Appendix S5 and Fig. S6) and counted the connections of all within- and between-canonical-network pairs. These counts were then normalized by the overall possible network-network connection counts (Greene et al., 2018; see Supplementary Appendix S6) which characterize the relative contribution of the canonical networks to the prediction of the future symptom severity (CBCL_y1_ in children and RCADS_y1_ in adolescents). Normalized count scores greater than 1 reflect canonical network pairs that are overrepresented in the prediction of symptom severity, that is, they contributed to prediction more than would be expected by chance.

**Fig. 5.**
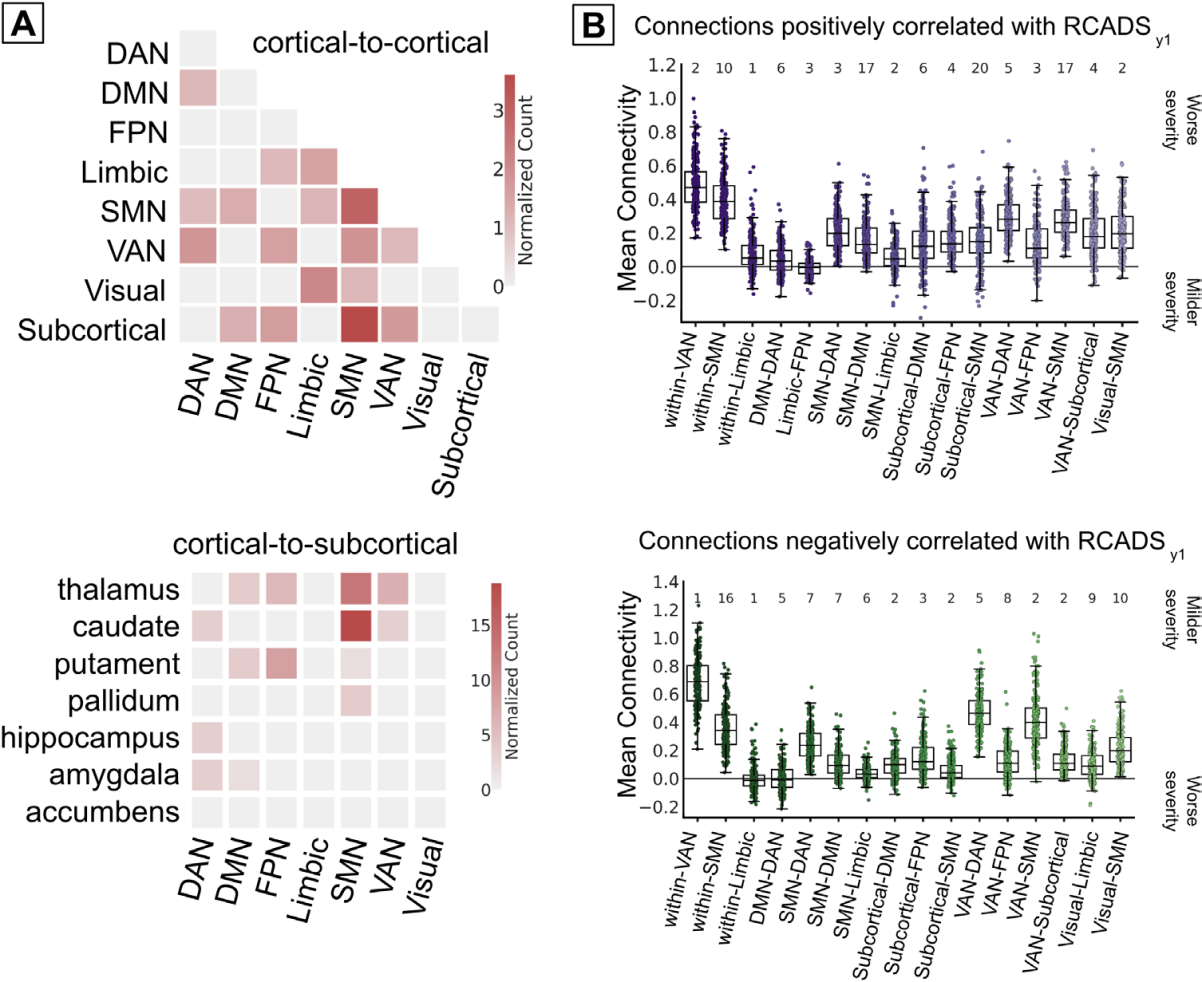
Network functional connectivity profiles characterization. (A) Normalized connection counts of overrepresented canonical cortical-to-cortical and cortical-to-subcortical pairs of the Symptoms Network. (B) Boxplots represent the distribution of mean FC values among connections from overrepresented network-network pairs, calculated for each participant separately. For interpretation purposes, we report separately the mean FC of the connections that were positively (top, purple) or negatively (bottom, green) correlated with RCADS_y1_. The numbers at the top of the graph represent the absolute connection counts of each network-network pair. For example, each value in the VAN-VAN distribution (panel B, purple) represents the mean FC of 10 connections for an individual participant. DAN: Dorsal Attention Network; DMN: Default Mode Network; FPN: Frontoparietal Network; SMN: Somatomotor Network; VAN: Ventral Attention Network.

The Symptoms Network (Fig. 5A) highlights three main patterns of (overrepresented) contributions involving sensory, attentional, and subcortical regions. The most numerous connections and most widely distributed patterns involved the SMN, involving every combination of network-network pairs, including within-SMN, between SMN to all other cortical networks, and SMN-to-subcortical regions. Notably, the only non-represented contributions were from SMN-FPN. Furthermore, there were numerous contributions involving the VAN, including within-VAN, between VAN or DAN to other cortical networks, and VAN-to-subcortical regions. Lastly, predictions relied on numerous connections involving subcortical regions which were more sparsely connected to associative areas (DMN, FPN, and VAN) and subcortical to SMN. The most numerous contributions in the subcortical-SMN connections involved the thalamus and basal ganglia (caudate, putamen, and pallidum; Fig. 5A, bottom).

Similar patterns are highlighted by the overall absolute, rather than normalized, network-network connection counts (Supplementary Fig. S7).

To better describe the FC profiles of the Symptoms Network in adolescents, we grouped the connections of the Symptoms Network into connections that were either positively or negatively correlated with RCADS_y1_ (representing brain-behavior associations at the group level). For each set of connections and network-to-network pair separately, we calculated the mean FC of each participant independently for the selected connections (representing FC profiles at the individual level). Figure 5B represents the within-participant FC distribution by network-to-network pairs, where each dot in the plot is the mean FC value of one participant.

The mean FC of the Symptoms Network was highly heterogeneous. In most network-to-network pairs, roughly half of the adolescents were characterized by positive FC and half by negative FC, that is, the participants’ FC were distributed around a mean connectivity of zero (e.g., see the FC distribution of within-limbic, DMN-DAN, limbic-FPN, SMN-limbic, and subcortical-SMN among others in Fig. 5B). Virtually every network-to-network comprised both connections that were either positively or negatively correlated with future symptom severity–that is, most network-to-network pairs are included in both Figure 5B top and bottom plots (e.g., see within-VAN). Furthermore, results were mixed also with respect to the association with future symptom severity—wherein strong FC of a network-to-network pair was associated with worse or milder symptom severity depending on the specific connection. For example, within-SMN connections were characterized by positive FC among all included adolescents whether positive FC was correlated with worse (Fig. 5B, top) or with mild (Fig. 5B, bottom) symptom severity.

However, some networks were characterized by similar FC patterns. Within-network connections of VAN-VAN, SMN-SMN, and limbic-limbic consistently displayed stronger positive FC, indicating cohesive activity patterns within each canonical functional network. With respect to subcortical connections (Supplementary Fig. S8), mean FC profiles showed similar heterogeneity wherein connections were mostly characterized by positive FC and every network-network pair comprised both positive and negative connections. However, as compared to other subcortical-to-cortical connections, the pallidum (to SMN) and amygdala (to DMN and to DAN) FC values were less spread, suggesting higher concordance between participants.

### 3.4 Extension of the symptoms network from ABCD to BANDA

The Symptoms Network, generated from ABCD, significantly predicted RCADS_y1_ in BANDA adolescents (Spearman’s rank correlation *ρ* = 0.236, *p* = 0.004, mean absolute error [MAE] = 9.40, root mean square error [RMSE] = 14.54). Critically, the prediction held even after partialling out the effect of RCADS_base_, sex at birth, age, and mean head motion (Spearman’s rank partial correlation *ρ* = 0.222, *p* = 0.007, MAE = 9.82, RMSE = 14.67; Supplementary Fig. S4).

### 3.5 Specificity of the symptoms network

We conducted specificity analysis for the Symptoms Network in both ABCD and BANDA datasets. In ABCD, specificity analyses (Supplementary Fig. S3) showed that the CBCL Anxious/Depressed subscale (i.e., the CBCL_y1_) was the CBCL subscale (out of 20 subscales) with the strongest correlation to the network strengths (i.e., FC) defined by the Symptoms Network. Note, differently from how we evaluated the performance and significance of the Symptoms Network, Supplementary Figure S3 reports the results of Spearman’s partial correlations generated on the full sample (n = 3,718) without employing cross-validation nor permutation testing. Thus, these are correlations and not predictions. As such, it was expected that the correlation (*ρ* = 0.198, Supplementary Fig. S3) would be inflated as compared to the predictions (*ρ* = 0.058), which were generated with a conservative approach.

In BANDA, specificity analyses revealed that RCADS_y1_ were the scores that the Symptoms Network predicted best among other self-reported measures of other psychopathology, cognitive, and general demographic measures (Supplementary Fig. S4), acquired as part of the full protocol for the BANDA study (for further details see Supplementary Appendix S3 and Hubbard et al., 2024). This possibly suggests that the Symptoms Network was sensitive to internalizing symptoms rather than reflecting a general vulnerability to psychopathology or other demographic characteristics. Furthermore, in-scanner head mean motion was not correlated with RCADS_base_ (*r* < 0.001, *p* = 0.987) but was negatively correlated with RCADS_y1_ (*r* = -0.02, *p* = 0.014; Supplementary Fig. S4). Quality control plots (Morfini et al., 2023) showed no evident bias in the connectivity estimates of each adolescent (Supplementary Fig. S5A) but suggested the presence of some residual effect of head motion at the group level (Supplementary Fig. S5B). Thus, the potential detrimental effect of motion-correlated FC was further minimized by adjusting all ABCD and BANDA analyses for mean head motion.

## 4 Discussion

Recent research has advanced the neural characterization of internalizing disorders in youth, but no research has investigated the generalizability of neural correlates of prospective symptom severity between childhood and adolescence. Addressing this gap, a CPM approach used in the childhood cohort (ABCD) identified a set of connections, which we term “Symptoms Network”, whose functional connectivity (FC) was related to individual differences in symptom severity at a 1-year follow-up. The Symptoms Network significantly predicted future symptom severity in children, correcting for baseline symptom severity, sex, age, and in-scanner mean head motion. Then, we demonstrated that symptom predictions derived from the Symptoms Network generalized to an independent sample of adolescents (BANDA) oversampled for internalizing disorders. Together, these results suggest that brain FC patterns associated with childhood depression and anxiety may persist during adolescence. Our results might also suggest that brain functional patterns associated with adolescent vulnerability to anxiety and depression may be identifiable earlier during childhood.

The Symptoms Network comprised distributed connections (Fig. 4), highlighting the contribution of multiple FC patterns, consistent with recent findings (Gracia-Tabuenca et al., 2024; He et al., 2021; Ho et al., 2022; Jin et al., 2020; Whitfield-Gabrieli et al., 2020). The contributing connections predominantly involved somatomotor, attention, and subcortical regions (Fig. 5), regions that have been previously investigated (largely by cross-sectional studies of affected adults) but that are overall less emphasized in the existing literature. Connections from the attentional and somatomotor networks might be indicative of heightened somatic awareness, hyper-sensitivity to bodily sensations even at rest, and states of ruminative or inward oriented attention, which often characterize anxious (Bouziane et al., 2022; MacNamara et al., 2016; Sylvester et al., 2013) and depressive conditions (Cui et al., 2024; Kaiser et al., 2015; Liu et al., 2019; Tse et al., 2024). Furthermore, numerous connections were found also within the SMN and between the SMN to frontal cortical networks (i.e., DMN, DAN, and VAN). The brain cortical development follows a sensorimotor-to-associative gradient, wherein sensory regions tend to mature earlier compared to associative areas (Baum et al., 2022; Chai et al., 2012; Gogtay et al., 2004; Sydnor et al., 2023). Our results might suggest that regions (i.e., SMN) which tend to reach within-network coherence earlier during the lifespan (Bethlehem et al., 2022; Gogtay et al., 2004) might already reflect certain signatures of vulnerability to internalizing disorders and may explain why these patterns can be identified as early as in childhood and even at rest.

Contrary to our hypotheses, predictive contributions in our model relied less than expected on the DMN, FPN, and other frontal or associative regions. These regions have been often linked to symptom severity in depression and anxiety via DMN hyperconnectivity or DMN-FPN dysregulation (Kaiser et al., 2015; Menon, 2011; Sheline et al., 2009) and are common targets of neuromodulatory interventions for depression (Mayberg et al., 2005; Siddiqi et al., 2020; Uddin et al., 2025; Zhang et al., 2023). Our results do not imply that the DMN, FPN, and other well-replicated findings did not contribute, nor are they unrelated to symptom severity or predictions in our model. Rather, we found that networks that undergo substantial reorganization during development (i.e., SMN and subcortical) may offer additional predictive value and may also be associated with future symptom severity. Furthermore, recent evidence suggests a key involvement in depression of the salience network (Lynch et al., 2024)—which overlaps with, but is not identical to, the FPN used here. Specifically, the salience network is expanded in depression, occupying a greater proportion of the cortex and encroaching regions that are assigned to adjacent networks in healthy controls. Our CPM approach selected connections regardless of network affiliation, yet our interpretation relied on a priori parcellation-based assignments. This may have assigned connections at the border of the FPN to adjacent networks instead, such as the VAN, which could potentially explain the importance of VAN-to-other network contribution to the prediction of future symptoms and the heterogeneity in FC profiles found within each set of connections. Lastly, as our model was trained on primarily healthy children, it may also have captured a distinct signature of future risk rather than signatures of active psychopathology. These findings suggest that risk and disorder expression may involve different circuit-level signatures, with implications for tailoring prevention interventions specifically more so than treatment.

Adolescents were characterized by substantial heterogeneity in their predictive FC profiles. Across most brain regions, mean FC values were widely-distributed among adolescents and centered around weak positive scores, in line with past findings (Goldstein-Piekarski et al., 2022; Kaiser et al., 2015; MacNamara et al., 2016), with a minority of adolescents being characterized by negative values (Fig. 5B; Supplementary Fig. S8). Virtually all network-to-network pairs (Fig. 5; Supplementary Fig. S7) included some connections whose stronger positive FC predicted more severe symptoms and some connections whose stronger positive FC predicted milder symptom severity. This pattern indicates that—on average across individuals—all identified connections contributed to the predictive model. However, the relevance to the prediction of each connection varied for every participant: a given connection might be a “protective” (i.e., associated with symptom improvement), “risk” (i.e., associated with symptom worsening), or neutral factor (i.e., marked by weak or near-zero association with symptom change), depending on the individual’s unique connectivity profile. This suggests that multiple, distinct neural mechanisms might contribute meaningfully to similar outcomes (Westlin et al., 2023), highlighting the potential shortcomings of uniform treatment approaches that do not account for individual differences. Such heterogeneity—where individuals with comparable symptom profiles may exhibit different neural correlates—ultimately underscores the need to develop interventions tailored to the individual and informed by each individual’s specific underlying mechanisms (Taxali et al., 2021).

The Symptoms Network predictions generalized from childhood to adolescence and were specific to anxiety and depression at both age ranges (Supplementary Figs. S3 and S4). This suggests the presence of common mechanisms associated with the psychopathology of both anxiety and depression, and at both age ranges. Anxiety and depression often co-occur; however, anxiety tends to emerge earlier in childhood (Kessler et al., 2005) and is often associated with homotypic and heterotypic trajectories with depression in adolescence. These data support the presence of an overlap between anxious and depressive states in adolescents (self-reported anxiety and depression correlations: baseline *r* = 0.822, *p* < 0.001; 1-year follow-up *r* = 0.806, *p* < 0.001) and underscores the challenge of uncoupling them (Auerbach et al., 2022). Testing the temporal mechanisms between anxiety and depression was beyond the scope of this study, but the generalizability of the Symptoms Network’s predictions from a community-based cohort (ABCD) to a more focused cohort oversampled for internalizing disorders (BANDA) could be taken as support for shared mechanisms underlying both anxiety and depression and highlights the importance of investigating the comorbidity between depression and anxiety.

Although both ABCD and BANDA are longitudinal observational studies, we observed a reduction in symptom severity between baseline and 1-year follow-up study visits in BANDA anxious-depressed participants, while ABCD and BANDA healthy participants reported consistently low and stable symptom levels. This reduction may reflect regression to the mean or natural recovery from more severe symptoms over time (Streiner, 2001). Our CPM model accounted for baseline symptom severity and included a healthy control group to mitigate such effects in predictive analyses (Yu & Chen, 2015), though they may still influence symptom distributions at individual timepoints. Given the episodic nature of depression, some participants may have been assessed during a symptom peak at baseline. Furthermore, informal or formal support may have contributed to symptom improvement. Importantly, this variability in symptom trajectories represents meaningful outcome patterns that predictive models must capture to provide added clinical value.

Nonetheless, the Symptoms Network predictions had modest effect sizes (*ρ* = 0.058 for children and *ρ* = 0.222 for adolescents), underscoring the challenge of translating neural correlates into clinical tools. The goal of the present study was not to identify a single, unified, and unique set of connections that underlie future risk of depression and anxiety, but rather to assess whether FC enclosed some information that represented an early sign of vulnerability to future psychopathology. Differently from other well-known descriptors of future risk of internalizing disorder–such as being female, young, and with a familial risk–FC can be considered a modifiable factor (Zhang et al., 2023) despite reliable configuration within individuals (Gordon et al., 2023) and with highly individualized topographic patterns (Finn et al., 2015). This study examined whether distributed patterns of FC associated with adolescent internalizing psychopathology could be identified in childhood–a time period that often precedes disorder onset. Our findings offer insights into the role of FC in mental health as it evolves throughout youth.

Our results suggest that FC improved symptom prediction. We took several analytical precautions to ensure the reliability, validity, generalizability, and specificity of our results. For reliability (Fig. 2A), we employed a leave-half-sites-out cross-validation technique within the ABCD cohort, as this approach is recommended for large samples (Poldrack et al., 2020; Scheinost et al., 2019) with a nested data structure (Rosenblatt et al., 2024). Since ABCD includes family members and multiple MRI sites, this ensured independence between splits of the cross-validation. We used permutation testing (Fig. 2B) to define results significance, reducing false positives linked with the large sample size of ABCD. For generalizability, we tested whether the ABCD-derived network could generate significant predictions in independent data from BANDA adolescents (Fig. 2C). To address skewed symptom scores (Fig. 3A), we used Spearman’s rank correlation and *t*-transformed scores for robust, interpretable results. Specificity was assessed by comparing the prediction for anxiety/depression severity with measures expected to be unrelated, in both children (Supplementary Fig. S3) and adolescents (Supplementary Fig. S4). We also controlled for baseline symptom severity, sex, age, and mean head motion, showing that our model detected effects beyond established predictors of future risk. Overall, this study afforded a novel framework that combines the advantages of large samples with those of specialized datasets. While large publicly available datasets are well powered to detect small heterogeneous effects, their broad scope limits the ability to detail specific phenomena of interest, afforded instead by usually smaller specialized datasets. By combining both approaches, results can capture community-wide effects while offering insights into targeted phenomena. Within this framework, the Symptoms Network represents a data-driven marker of early vulnerability–trained on mostly healthy children yet predictive in both healthy and clinically affected adolescents. Detecting signatures of psychopathology risk in mostly healthy individuals and validating them in individuals experiencing anxiety or depression poses a significant yet crucial challenge for mental health research. Achieving added predictive performance despite these factors is critical for advancing early identification and prevention strategies, highlighting the potential value for real-world applications of biological markers (Woo et al., 2017).

### 4.1 Limitations

There are several noteworthy limitations. First, the datasets were not the same age ranges, and thus, not a direct validation of the ABCD-derived Symptoms Network. However, applying cross-validation internally to a large sample, such as ABCD, allows training and testing on well-powered subsamples of ~1,860 children (Garavan et al., 2018), guarding against overfitting. Second, while we referred to 9–10 years-old participants from ABCD as children, these ages could also be considered late childhood or early adolescence. Third, CPM tested for linear effects over time; however, it is plausible that symptom development could be characterized by non-linear trends. Fourth, although including all the possible functional connections in our CPM approach is consistent with standard practice in the field (Kucyi et al., 2021; Shen et al., 2017; Taxali et al., 2021), this high-dimensional feature space may reduce model precision due to multicollinearity and individual variability. Although this comprehensive modeling strategy captures the full connectome and preserves potential predictive signals, it may also contribute to higher rate of false negative errors and underestimation of true effect sizes. Fifth, although the prediction effect sizes were modest, they are in line with those reported in prior studies employing similar methodologies (Marek et al., 2022). It is noteworthy that unlike most studies, which examine the associations between brain connectivity and concurrent symptoms, the Symptoms Network predictions were further penalized by the challenge of predicting future severity (rather than concurrent). This prediction was further constrained by statistical correction for baseline severity, which itself was strongly correlated with 1-year follow-up severity across participants (Supplementary Fig. S2). These factors underscore the robustness of the observed predictive patterns despite the small effect sizes. Furthermore, small shifts in the effect size have been shown to impact disproportionally more the extreme cases of a distribution (Carey et al., 2023). In the case of mental health, these would be the individuals suffering from depression and anxiety, for example. For those, even small effect sizes may be both meaningful and impactful, especially considering the compounded effects of early prevention or interventions over time.

## 5 Conclusions

CPM identified FC patterns that predicted future depression and anxiety severity across independent samples of children and adolescents. Distributed connectivity patterns of attentional, sensorimotor, and subcortical systems contributed to the prediction of symptom severity at a 1-year follow-up assessment, suggesting that brain FC may meaningfully contribute to informing future depression and anxiety severity. Accordingly, neural networks may provide targets for the development and testing of treatments for youth (e.g., real-time neurofeedback, transcranial magnetic stimulation). However, the modest effect sizes and heterogeneity of results, as found in our study and other prior work, highlights the challenges of translating brain-based correlates into clinical tools and suggests that such correlates may not apply uniformly across individuals. Ultimately, our results point to the potential value of employing personalized approaches tailored to individual neurobiological profiles in youth.

## Supplementary Material

Supplementary Material

## Data Availability

Publicly available datasets were analyzed in this study which can be found in the National Institute of Mental Health Data Archive data including *Connectomes Related to Anxiety and Depression in Adolescents (BANDA)* collection #3037 and *DCAN Labs ABCD-BIDS Community Collection (ABCC)* #3165. Analysis code for the current project can be found at https://github.com/fmorfini/publications/tree/main/CPM_ABCD_BANDA_predict_future_depression_anxiety
