# Supplementary material for: Brain functional connectivity predicts depression and anxiety during childhood and adolescence: A connectome-based predictive modeling approach
Source: Imaging Neurosci (Camb). 2025 Sep 12;3:IMAG.a.145. doi: 10.1162/IMAG.a.145 (PMC12434380; doi:10.1162/IMAG.a.145)
Supplement: Supplementary Material [file IMAG.a.145_supp.pdf]

Supplementary Information

Brain functional connectivity predicts depression and anxiety during childhood and adolescence: A connectome-based predictive modeling approach

Francesca Morfini, MS[1,*], Aaron Kucyi, PhD[2], Jiahe Zhang, PhD[1,3], Clemens C. C. Bauer, PhD[1,3,4], Paul A. Bloom, PhD[5,6], David Pagliaccio, PhD[5,6], Nicholas A. Hubbard, PhD[7], Isabelle M. Rosso, PhD[8,9], Anastasia Yendiki, PhD[10], Satrajit S. Ghosh, PhD[4], Diego A. Pizzagalli, PhD[8,9], John D.E. Gabrieli, PhD[4], Susan Whitfield-Gabrieli, PhD[1,3,4,10], & Randy P. Auerbach, PhD, ABPP[5,6]

[1]Department of Psychology, Northeastern University, Boston, MA, USA
[2]Department of Psychological and Brain Sciences, Drexel University, Philadelphia, PA, USA
[3]Center for Precision Psychiatry, Massachusetts General Hospital, Boston, MA, USA
[4]Department of Brain and Cognitive Sciences and McGovern Institute for Brain Research, Massachusetts Institute of Technology, Cambridge, MA, USA
[5]Department of Psychiatry, Columbia University, New York, NY, USA
[6]Division of Child and Adolescent Psychiatry, New York State Psychiatric Institute, Columbia University, New York, NY, USA
[7]Department of Psychology, University of Nebraska-Lincoln, Lincoln, NE, USA
[8]Center for Depression, Anxiety, and Stress Research, McLean Hospital, Belmont, MA, USA
[9]Department of Psychiatry, Harvard Medical School, Boston, MA, USA
[10]Athinoula A. Martinos Center for Biomedical Imaging, Massachusetts General Hospital, Charlestown, MA, USA

DOI: https://doi.org/10.1162/IMAG.a.145

---

* Correspondence: Francesca Morfini, Department of Psychology, Center for Cognitive and Brain Health, Northeastern University, 805 Columbus Avenue, Boston, MA 02120, United States; Email: f.morfini.work@gmail.com

Table of Contents

Appendix S1. ABCD dataset description.

Appendix S2. ABCD MRI data preprocessing.

Appendix S3. BANDA dataset description.

Appendix S4. BANDA MRI data preprocessing.

Appendix S5. Mapping of Gordon parcellation to Yeo 7 canonical brain networks.

Appendix S6. Brain functional networks relative contribution to symptoms prediction.

Table S1. Participants, clinical, and scanner characteristics of the ABCD children.

Table S2. Participants, clinical, and scanner characteristics of the BANDA adolescents.

Table S3. BANDA clinical characteristics.

Table S4. RCADS subscales pairwise correlations in adolescents from BANDA.

Figure S1. CONSORT diagram of included and excluded participants.

Figure S2. Symptom severity and in-scanner head mean motion correlation.

Figure S3. Internal validation and predictions specificity in ABCD.

Figure S4. External validation and predictions specificity from ABCD to BANDA.

Figure S5. fMRI quality control distributions in BANDA.

Figure S6. Gordon parcellation assignment to Yeo 7 canonical networks.

Figure S7. Absolute network-network counts of the Symptoms Network.

Figure S8. Within-participant between-connections mean functional connectivity of the subcortical-to-cortical connections of the Symptoms Network in BANDA.

Supplementary References

Appendix S1. ABCD dataset description.

Sample characteristics and study design

ABCD is a public and longitudinal study from 21 USA sites aimed at characterizing neural, cognitive, and behavioral development in a large population (N = 11,875) of children of diverse race, ethnicity, education, environmental, and income levels who are 9-10-years-old at baseline. Each site received Institutional Review Board approval from their institution and written informed consent and assent were received by the guardian and participating children. Recruitment of children and their guardians was carried out through schools and community settings. A description of the recruitment, inclusion, and exclusion criteria, and study design has been detailed elsewhere (Garavan et al., 2018).

In this study, we included data from the baseline and 1-year follow-up visits. At each study visit, the MRI and clinical assessments typically happened on the same day. Anatomical and functional MRI data underwent a live quality control during data acquisition by scan operators, as well as both automated and visual inspection, including detection of excessive in-scanner head motion, signal to noise inhomogeneities, and anatomical abnormalities (Chai et al., 2012). Reasons for participant exclusion included in-scanner high motion, talking, falling asleep, using the safety squeeze ball to communicate with the operator, or interrupted scanning, and these reasons were coded into a pass or fail information. At both baseline and 1-year follow-up assessments, a guardian reported on their child's depression and anxiety severity using the Child Behavior Checklist (CBCL) Anxious/Depressed subscale (Achenbach, 1991).

We used data from the ABCD-BIDS Community Collection (**Figure S1A)**. This collection processed and analyzed only data from ABCD which had passed the quality control carried out by the consortia (excluded 2,471 participants). Additionally, we excluded participants

with missing CBCL Anxious/Depressed subscale *t*-transformed scores at either the baseline ($CBCL_{base}$) or 1-year follow-up ($CBCL_{y1}$) assessment (excluded additional 515 participants). We also excluded one of the family members of families that were scanned at different sites (of 10 families we excluded 5 participants, selecting the family member scanned at the site that had the biggest sample size out of the two sites). Lastly, exclusion criteria included having high an in-scanner mean head motion (FD > 0.25 mm) and less than 10 minutes of overall fMRI data after removing outliers. Overall, 3,718 participants were included in our study.

Depression and anxiety symptoms assessment

The Child Behavior Checklist (CBCL; Achenbach, 1991) is a 118-item caregiver report of child behavioral characteristics based on a 3-point Likert scale between 0 (*not true*) and 2 (*very true*). The subscale of interest was the Anxious/Depressed subscale based on 13 items with a range of possible scores between 0 and 26. Higher scores indicate greater symptom severity. The *t*-transformed scores were used. The Cronbach's alpha for our sample was 0.825 with a 95% confidence interval (CI) of [0.816, 0.833] at baseline and 0.818 with 95% CI [0.809, 0.826] at the 1-year assessment.

MRI data

MRI data were acquired at 3T via Siemens, General Electric, and Philips MRI scanners (**Table S1**) using multiband echo planar imaging (EPI) acquisition. MRI sequences were harmonized across scanners types and across sites, which had been in turn built starting from the Human Connectome Project (HCP) sequences (Smith et al., 2013). Imaging data characteristics are detailed elsewhere (Casey et al., 2018). Briefly, anatomical MRI data were T1-weighted multi-

echo magnetization-prepared rapid gradient echo (MPRAGE). Resting-state fMRI (rs-fMRI) data were four 5-min runs during which participants were instructed to passively gaze at a cross hair. The anatomical scans were acquired first, followed by the rs-fMRI runs.

Appendix S2. ABCD MRI data preprocessing.

Baseline rs-fMRI data were accessed from the ABCD-BIDS Community Collection 3165 (Feczko et al., 2021) as fully preprocessed, quality-controlled, data in the form of connectomes. Preprocessing details can be found under the Collection's online documentation (https://collection3165.readthedocs.io/en/stable/pipeline/). Briefly, the data had been processed using a modified version of the HCP pipeline (Glasser et al., 2013) customized and applied by the DCAN Lab (Feczko et al., 2021). Rs-fMRI data were registered onto the Montreal Neurological Institute (MNI) standard space using a combination of surface and volume coordinate systems. Preprocessed rs-fMRI data were then further denoised by removing signal factors, including head motion, mean time series for white matter, cerebrospinal fluid, and global signal, as well as movement factors such as realignment parameters. All frames with framewise displacement (FD) < 0.2 mm (Chai et al., 2012) were retained and used for functional connectivity estimation.

Preprocessed data were parcellated following a cortical (Gordon et al., 2016) and subcortical (Fischl et al., 2002) schema, band-pass filtered (0.008, 0.09 Hz), and concatenated. Then, the time series of each parcel were extracted, and Pearson's correlated to the time series of every other parcel, and Fisher r-to-z-transformed. This process resulted in the construction of one symmetrical 352-by-352 connectivity matrix (i.e. connectome) per participant, representing their whole-brain functional connectivity patterns (**Figure 1** from the main text).

Appendix S3. BANDA dataset description.

Sample characteristics and study design

Information of the study protocol and information of the full sample for the Boston Adolescent Neuroimaging of Depression and Anxiety (BANDA) dataset, also referred to as Human Connectomes Project for Disease related to Anxiety and Depression in Adolescents, are described elsewhere (Hubbard et al., 2020, 2024; Siless et al., 2020). Briefly, participants were included if ages between 14 and 17 years-old at the baseline assessment and both participant and their guardian were fluent in English. Exclusion criteria for the participants were: unable to undergo MRI scanning, presenting with IQ < 85, having any neurodevelopmental disorders, bipolar disorder, psychotic disorder, premature birth, serious medication conditions, history of serious head injury, or hospitalization following a neurological or cardiovascular disease.

Clinical assignment included the Kiddie Schedule for Affective Disorders and Schizophrenia Present and Lifetime Version (K-SADS; Kaufman et al., 1997) adapted to provide DSM-5 compatible classifications. Depressed-anxious participants were defined as those presenting at baseline with a primary diagnosis of at least one depressive (i.e., major depressive disorder, dysthymia, or depression not otherwise specified) and/or anxiety disorders (i.e., generalized anxiety disorder, social phobia, separation anxiety, panic disorder, agoraphobia, or specific phobia). Presenting with a diagnosis of attention-deficit/hyperactivity disorder (ADHD), which is highly comorbid with depression and anxiety, and/or being on psychotropic medications were not considered exclusion criteria for the depressed-anxious group (for further rationale see Hubbard et al., 2020, 2024). Healthy controls were defined as those without any lifetime history of psychiatric disorders at baseline.

All adolescents included in the current study participated in at least two study visits, one year apart. The baseline visit included an MRI scanning session, a diagnostic interview, collection of self-report, collection of parental measures of symptom severity including the Revised Child Anxiety and Depression Scale (RCADS; de Ross et al., 2002), and neurocognitive assessments. During the 1-year follow-up assessment, symptom severity was assessed again. As part of the full protocol for the BANDA study (Hubbard et al., 2020, 2024), a number of other measures of psychopathology, cognitive performance, and general demographic information were acquired at both baseline and 1-year follow-up assessments via the following: Behavioral Inhibition and Behavioral Activation Questionnaire (BISBAS; Carver & White, 1994), Chapman Handedness Inventory (Chapman & Chapman, 1987), Columbia Suicide Severity Rating Scale (CSSRS; Posner et al., 2011), Mood and Feelings Questionnaire (MFQ; Angold et al., 1995), Risky Behavior Questionnaire for Adolescents (RBQA; Auerbach & Gardiner, 2012), Snaith-Hamilton Pleasure Scale (SHAPS; Carver & White, 1994), State-Trait Anxiety Inventory (STAI; Spielberger et al., 1970), Wechsler Abbreviated Scale of Intelligence (WASI-II; Wechsler, 2018), in addition to the RCADS.

In this study, participants were excluded if there were incomplete data consisting of baseline anatomical, rs-fMRI data, or completion of the RCADS at both the baseline ($RCADS_{base}$) and 1-year assessment ($RCADS_{y1}$). Participants were excluded also if the in-scanner mean head motion was FD > 0.25 mm. The final sample included 150 adolescents (see **Table S2** for the sample's demographic and clinical information; **Figure S1B** for a flowchart of the included and excluded participants).

Depression and anxiety symptom assessment

Depression and anxiety severity was assessed with the Revised Child Anxiety and Depression Scale (RCADS; de Ross et al., 2002). The RCADS total scores were calculated as the sum of the 47 items rated on a 4-point Likert scale between 0 (*never*) and 3 (*always*) and represented depression and anxiety severity. RCADS total scores can range between 0 and 141, with higher scores representing greater symptom severity. The RCADS includes the following subscales: social phobia, panic disorder, major depression, separation anxiety, generalized anxiety, and obsessive-compulsive disorder. A measure of self-reported anxiety severity can be calculated by summing all the subscales except for the major depression subscale. The *t*-transformed scores were used. The Cronbach's alpha for the RCADS total scores of our sample was 0.970 with 95% CI [0.964, 0.977] at baseline and 0.963 with 95% CI [0.954, 0.971] at the 1-year assessment.

MRI data

The description of MRI sequences, protocols, and harmonization with other HCP datasets are more extensively reported elsewhere (Siless et al., 2020; Tozzi et al., 2020). Briefly, the MRI data were collected on a Siemens 3T Prisma MRI with a 64-channel head coil. One high resolution anatomical image was acquired with a T1-weighted MPRAGE sequence (0.8 mm isotropic voxels, field of view = 256 x 240 x 167 mm, TR = 2,400 ms, TE = 2.18 ms). rs-fMRI were acquired using simultaneous multi-slice (2.0 mm isotropic voxels, 72 slices, multiband acceleration factor = 8, TR = 800 ms, TE = 37 ms, flip angle = 52°), each consisting of 420 volumes lasting 5 min and 46 s. Two sets of two eyes-open rs-fMRI (four total runs lasting overall ~23) with opposite phase encoding (PE) direction (Anterior-Posterior [AP] and Posterior-

Anterior [PA]) were acquired and were alternated with the acquisition of a Spin-Echo fieldmap with opposite PE direction (AP-PA).

Appendix S4. BANDA MRI data preprocessing.

BANDA connectomes generation

After preprocessing and denoising, anatomical and functional data were parcellated, denoised, and organized into connectomes using the CONN Toolbox (Whitfield-Gabrieli & Nieto-Castanon, 2012). FMRI data were parcellated into 333 cortical (Gordon et al., 2016) and 19 subcortical regions (Fischl et al., 2002). Connectomes were generated by calculating the Fisher-z transform of the Pearson *r* correlation coefficient between the mean time series of each of the 352 regions to the time series of every other region. This step resulted in one, symmetrical connectome per participant and mimicked the connectome constructions carried out for the ABCD participants.

Appendix S5. Mapping of Gordon parcellation to Yeo 7 canonical brain networks.

For interpretation purposes, each of the regions from the Gordon parcellation was mapped onto one of the 7 Yeo networks (Yeo et al., 2011) by calculating the spatial overlap of each parcel to the Yeo's networks liberal masks, both in MNI volume space, using the CONN v.21a (Whitfield-Gabrieli & Nieto-Castanon, 2012) in-built conn_roioverlaps function. Briefly, each voxel forming a parcel would be assigned to one Yeo network. The Yeo network with the highest count of voxels in a parcel and a total match count of at least 50 voxels would define which network that parcel would be assigned to. Five of the 333 Gordon's parcels were not assigned to any of Yeo's networks (voxels overlap = 19.40 ± 23.90, range [1, 48]).

Appendix S6. Brain functional networks relative contribution to symptoms prediction.

To characterize the relative contribution of each canonical network pair to the prediction of prospective symptom severity, we calculated counts of connections of all within- and between-networks pairs from the Symptoms Network and normalized them by their total possible size (Greene et al., 2018), as follows:

$$\text{Contribution score}_{AB} = \frac{\dfrac{Surviving\ Edges_{AB}}{Surviving\ Edges_{total}}}{\dfrac{Edges_{AB}}{Edges_{total}}}$$

where A and B represent two canonical networks; $Surviving\ Edges_{AB}$ / $Surviving\ Edges_{total}$ represents the ratio between the surviving connections between two canonical networks (A and B) over all the surviving connections (n = 251 unique connections)); and the denominator $Edges_{AB}$ / $Edges_{total}$ represents the ratio between all possible connections between network A and network B over all possible connections of the chosen parcellation, i.e., [352 * (352 -1)] / 2 = 61,776 connections . Contribution scores above 1 identify canonical network pairs that are overrepresented in our Symptoms Network relative to their possible size.

Table S1. Participants, clinical, and scanner characteristics of the ABCD children.

ABCD dataset	Included participants (n = 3,718)
Sex [n, %]	
Male	1,967 (52.90%)
Female	1,751 (47.10%)
Race [n]	
White	3,136
Black or African American	494
Asian	264
American Indian Native American	123
Hawaiian or Pacific Islander	23
Others	171
Unknown or not reported	24
Ethnicity [n]	
Not Hispanic or Latino	3,074
Hispanic or Latino	606
Do not know, refuse to answer, NA	38
Age (months) [n]	
Baseline	120.16 (±7.49)
1-Year Follow-Up	132.33 (±7.7)
CBCL Anxious/Depressed raw score	
Baseline	2.54 (±3.12)
1-Year follow-up	2.59 (±3.1)
CBCL Anxious/Depressed *t*-score	
Baseline	53.48 (±6.06)
1-Year follow-up	53.51 (±6.02)
Scanner manufacturer [n]	
SIEMENS	2,640
General Electric Medical Systems	832
Philips Medical Systems	246
Scanner Model [n]	
Prisma	1,359
Prisma_fit	1,281
DISCOVERY MR750	793
Achieva dStream	149
Ingenia	97
SIGNA Creator	39
In-scanner mean motion (mm)	0.16 (±0.05)

Characteristics are presented for the participants included in our study (n = 3,718). Values are reported as mean (± standard deviation), unless otherwise specified. ABCD: Adolescent Brain Cognitive Development; CBCL: Child Behavior Checklist.

Table S2. Participants, clinical, and scanner characteristics of the BANDA adolescents.

BANDA dataset	Healthy (n = 54)	Depressed-Anxious (n = 96)	t|χ2	p
Sex [n]			0.84	0.36
Female	30	62		
Male	24	34		
Age (months)				
Baseline	182.94 (±9.91)	186.62 (±10.43)	-2.11	0.04 *
1-Year follow-up	195,85 (±10.26)	199.8 (±10.43)	-2.24	0.03 *
Race [n]			1.31	0.93
White	43	77		
More than one race	7	14		
Asian	2	2		
Black or African American	1	1		
Hawaiian or Pacific Islander	0	1		
Unknown or not reported	1	1		
Ethnicity [n]			1.14	0.57
Not Hispanic or Latino	50	87		
Hispanic or Latino	4	7		
Unknown or not reported	0	2		
RCADS total raw score				
Baseline	14.17 (±9.46)	50.26 (±24.13)	-10.53	<0.001 ***
1-Year follow-up	15.46 (±12.08)	39.53 (±21.45)	-7.59	<0.001 ***
RCADS total *t*-score				
Baseline	36.04 (±4.43)	56.47 (±14.26)	-10.24	<0.001 ***
1-Year follow-up	36.7 (±5.25)	49.07 (±11.5)	-7.47	<0.001 ***
In-scanner mean motion (mm)	0.1 (±0.04)	0.1 (±0.04)	-0.21	0.83

Characteristics are presented for the participants included in our study (n = 150). Values are reported as mean (± standard deviation), unless otherwise specified. The depressed-anxious group includes participants presenting at baseline with at least a primary depressive and/or anxiety disorder diagnosis, whereas healthy participants had no current or lifetime history of DSM-5 mental disorders at the baseline assessment. Comparisons between the depressed-anxious and healthy participants represent either statistics from independent-sample t-test or Chi square test. BANDA: Boston Adolescent Neuroimaging of Depression and Anxiety; RCADS: Revised Child Depression and Anxiety Scale; *: $p < 0.05$; ***: $p < 0.001$.

Table S3. BANDA clinical characteristics.

Depressed-Anxious BANDA participants	Baseline (n = 96)	1-Year Follow-Up (n = 71)
**DSM-5 depressive disorders**		
Major depressive disorder	38	18
Dysthymia	2	5
Depression not otherwise specified	0	0
**DSM-5 anxiety disorders**		
Generalized anxiety disorder	51	35
Social phobia	46	39
Separation anxiety	6	1
Panic disorder	11	8
Agoraphobia	5	2
Specific phobia	23	16
**DSM-5 other disorders**		
Attention-deficit/hyperactivity disorder	27	20
Obsessive-compulsive or related disorders	11	8
Post-traumatic stress disorder	2	0

Presence of diagnoses reported in the table were based on the Kiddie Schedule for Affective Disorders and Schizophrenia Present and Lifetime Version (Kaufman et al., 1997) clinical interview adapted to assess DSM-5 (American Psychiatric Association, 2013) mental disorders. The table reports current diagnoses at each study assessment. DSM-5: Diagnostic and Statistical Manual of Mental Disorders 5th edition.

Table S4. RCADS subscales pairwise correlations in adolescents from BANDA.

		Baseline			1-year follow-up		
**Subscale1**	**Subscale2**	**r**	**CI 95%**	**p-corrected**	**r**	**CI 95%**	**p-corrected**
Total	Social phobia	0.8753	[0.83 0.91]	<0.0001	0.8859	[0.85 0.92]	<0.0001
Total	Panic disorder	0.8977	[0.86 0.92]	<0.0001	0.8368	[0.78 0.88]	<0.0001
Total	MDD	0.8953	[0.86 0.92]	<0.0001	0.8942	[0.86 0.92]	<0.0001
Total	GAD	0.9091	[0.88 0.93]	<0.0001	0.8901	[0.85 0.92]	<0.0001
Total	OCD	0.7669	[0.69 0.83]	<0.0001	0.6647	[0.56 0.75]	<0.0001
Total	SAD	0.7899	[0.72 0.84]	<0.0001	0.7796	[0.71 0.84]	<0.0001
Total	Total anxiety	0.9895	[0.99 0.99]	<0.0001	0.9856	[0.98 0.99]	<0.0001
Social phobia	Panic	0.7205	[0.63 0.79]	<0.0001	0.6865	[0.59 0.76]	<0.0001
Social phobia	MDD	0.7053	[0.61 0.78]	<0.0001	0.7208	[0.63 0.79]	<0.0001
Social phobia	GAD	0.7558	[0.68 0.82]	<0.0001	0.7612	[0.68 0.82]	<0.0001
Social phobia	OCD	0.5663	[0.45 0.67]	<0.0001	0.4332	[0.29 0.55]	<0.0001
Social phobia	SAD	0.6061	[0.49 0.70]	<0.0001	0.6251	[0.52 0.71]	<0.0001
Social phobia	Total anxiety	0.8914	[0.85 0.92]	<0.0001	0.9001	[0.86 0.93]	<0.0001
Panic disorder	MDD	0.7552	[0.68 0.82]	<0.0001	0.7394	[0.66 0.80]	<0.0001
Panic disorder	GAD	0.7777	[0.71 0.83]	<0.0001	0.6494	[0.55 0.73]	<0.0001
Panic disorder	OCD	0.6334	[0.53 0.72]	<0.0001	0.4199	[0.28 0.54]	<0.0001
Panic disorder	SAD	0.7023	[0.61 0.78]	<0.0001	0.5746	[0.46 0.67]	<0.0001
Panic disorder	Total anxiety	0.904	[0.87 0.93]	<0.0001	0.8281	[0.77 0.87]	<0.0001
MDD	GAD	0.7786	[0.71 0.83]	<0.0001	0.7298	[0.64 0.80]	<0.0001
MDD	OCD	0.6596	[0.56 0.74]	<0.0001	0.5137	[0.39 0.62]	<0.0001
MDD	SAD	0.6346	[0.53 0.72]	<0.0001	0.5815	[0.46 0.68]	<0.0001
MDD	Total anxiety	0.8215	[0.76 0.87]	<0.0001	0.8058	[0.74 0.86]	<0.0001
GAD	OCD	0.7191	[0.63 0.79]	<0.0001	0.6598	[0.56 0.74]	<0.0001
GAD	SAD	0.7154	[0.63 0.79]	<0.0001	0.7383	[0.66 0.80]	<0.0001
GAD	Total anxiety	0.911	[0.88 0.93]	<0.0001	0.9023	[0.87 0.93]	<0.0001
OCD	SAD	0.5645	[0.44 0.66]	<0.0001	0.6327	[0.53 0.72]	<0.0001
OCD	Total anxiety	0.7676	[0.69 0.83]	<0.0001	0.6855	[0.59 0.76]	<0.0001
SAD	Total anxiety	0.8051	[0.74 0.86]	<0.0001	0.812	[0.75 0.86]	<0.0001

Pairwise Pearson correlations among Revised Child Anxiety and Depression Scale (RCADS) subscales at the baseline and 1-year follow-up assessments for the BANDA adolescents (n = 150). RCADS subscales included generalized anxiety (GAD), major depressive disorder (MDD), obsessive-compulsive disorder (OCD), panic disorder, separation anxiety disorder (SAD), and social phobia. Total Anxiety reflects the sum of all subscales except for MDD, whereas Total includes all subscales. *P* values are two-sided and Bonferroni-corrected for multiple comparisons.

Figure S1. CONSORT diagram of included and excluded participants.

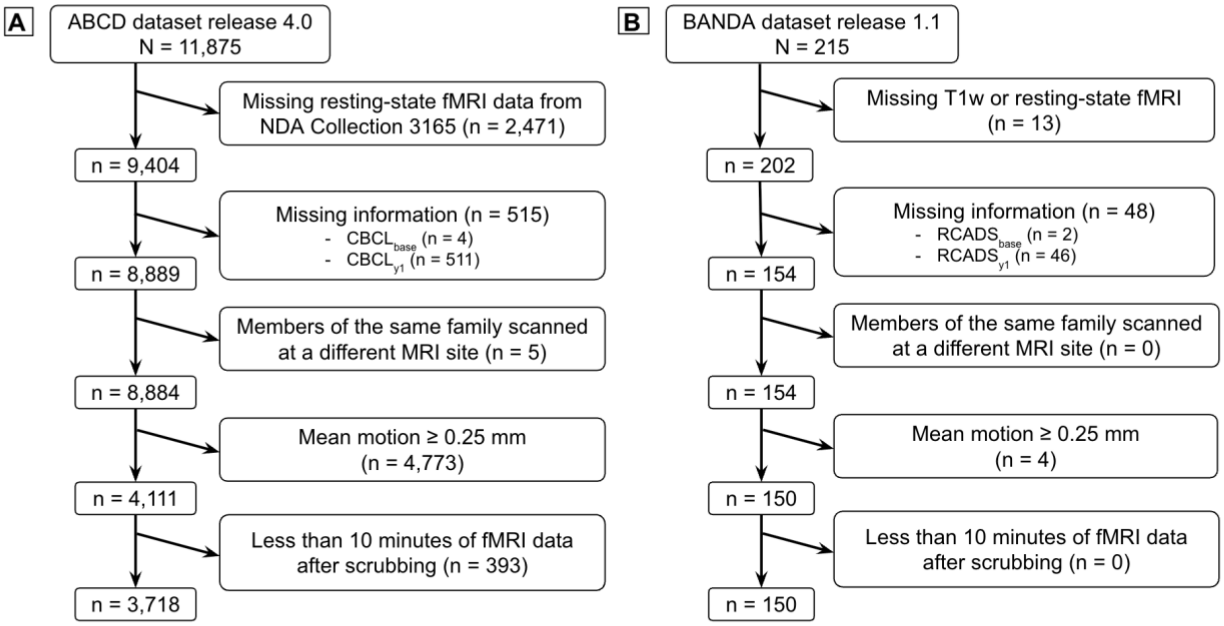

(**A**) Flow of exclusion for ABCD participants. rs-fMRI data were obtained from the fully preprocessed ABCD-BIDS Community Collection 3165 (Feczko et al., 2021) which only included data from the Annual Curated ABCD 4.0 Data Release (Barch et al., 2018) that had passed the Data Analysis Imaging Center quality control (Chai et al., 2012). (**B**) Flow of exclusion for BANDA participants. ABCD: Adolescent Brain Cognitive Development Study; BANDA: Boston Adolescent Neuroimaging of Depression and Anxiety; CBCL: Child Behavior Checklist, Anxious/Depressed subscale raw scores; fMRI: functional magnetic resonance imaging; NDA: National Institute of Mental Health Data Archive; RCADS: Revised Child Depression and Anxiety Scale, total raw scores.

 https://doi.org/10.1162/IMAG.a.145

Figure S2. Symptom severity and in-scanner head mean motion correlation.

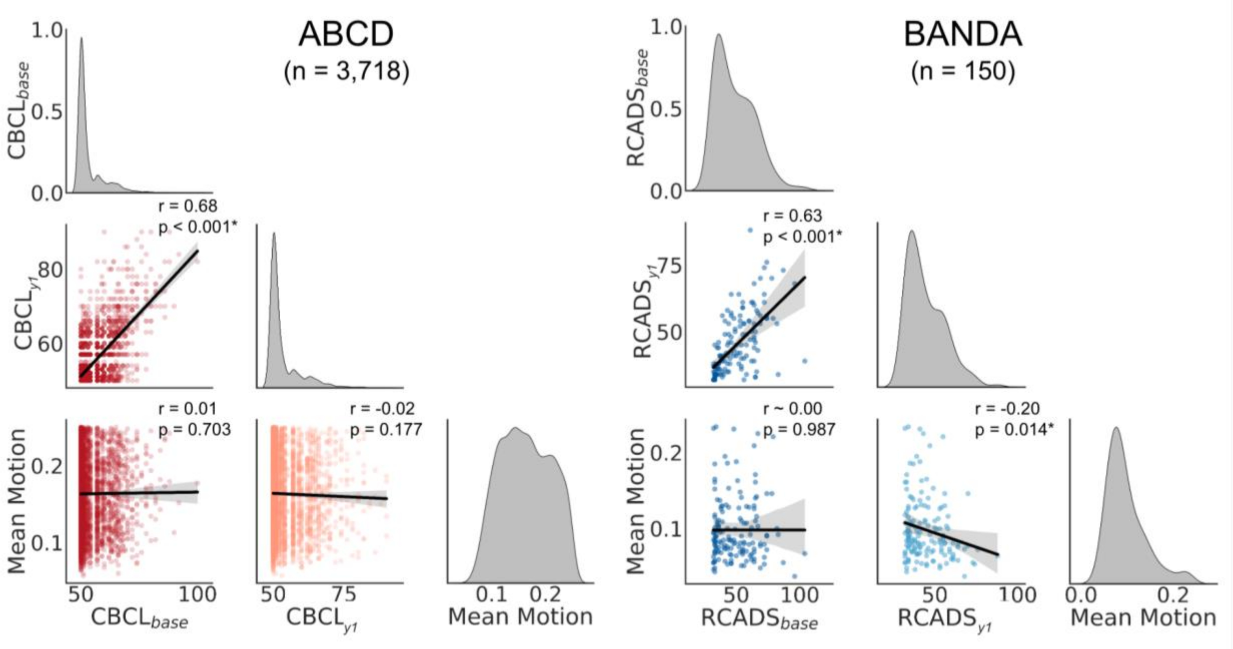

The scatterplots represent Pearson's correlations coefficients *r* between depression and anxiety symptom severity at the baseline and 1-year follow-up assessments with in-scanner head mean motion (framewise displacement in mm Chai et al., 2012) for the included participants of the ABCD (left) and BANDA cohorts (right). Symptom severity was calculated using the CBCL Anxious/Depressed subscale *t*-transformed scores in ABCD and using the RCADS *t*-transformed total scores in BANDA, respectively. The plots report the 99% confidence interval (shaded areas). ABCD: Adolescent Brain Cognitive Development Study; BANDA: Boston Adolescent Neuroimaging of Depression and Anxiety; base: baseline assessment; CBCL: Child Behavior Checklist Anxious/Depressed subscale, *t*-transformed scores; CPM: connectome-based predictive modeling; y1: 1-year follow-up assessment; RCADS: Revised Child Depression and Anxiety Scale, *t*-transformed total scores. *: $p < 0.001$.

Figure S3. Internal validation and predictions specificity in ABC

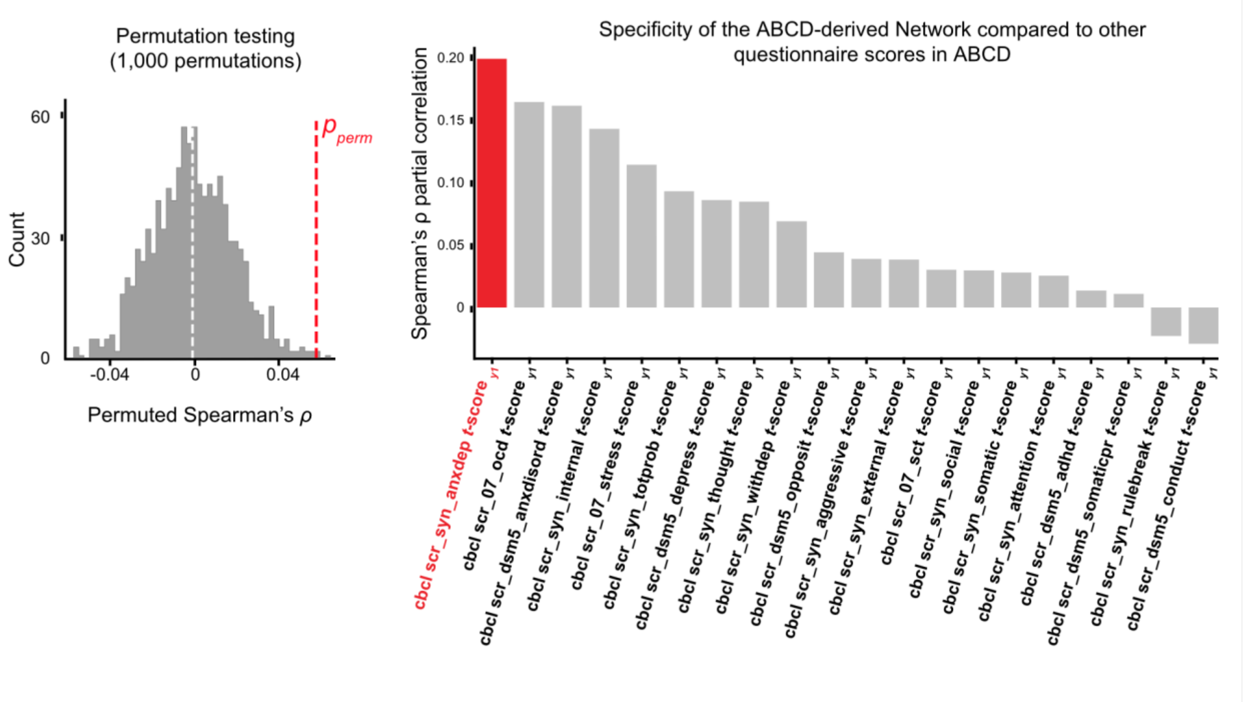

(**Left**) Null distribution of ρ values generated from 1,000 permutations derived with shuffled data (i.e., connectivity values of one participant were used to predict the symptom severity of another random participant). The permuted distribution is centered around $\rho = 0$ (white dashed line). Negative ρ values represent cases where the predictions were inaccurate, for example mild symptoms were predicted but severe symptoms were observed. Network significance (p_{perm}, red dashed line) was defined as the proportion of permuted ρ values larger than the ρ value (average of 100 iterations) generated by the observed data (Shen et al., 2017). (**Right**) To evaluate the specificity of the Symptoms Network to anxiety and depression in the ABCD children, we computed Spearman's partial correlation with each of all CBCL subscales as outcome (adjusting for baseline $CBCL_{base}$, sex, age, and mean head motion). Note, the results shown here differ from those reported in the main text (e.g., for $CBCL_{y1}$, red bar), in that here we use the Networks Symptoms to extract network strengths from participants, we use Spearman's partial correlation and not cross-validation and permutation testing, and we used the full sample (n = 3,718). Consequently, the bar graph does not represent predictions, rather in-sample correlations. Nonetheless, the correlation estimates for the different CBCL subscales offer a comparison between the performance of Symptoms Network for anxiety and depression as compared to other symptoms.

Figure S4. External validation and predictions specificity from ABCD to BANDA.

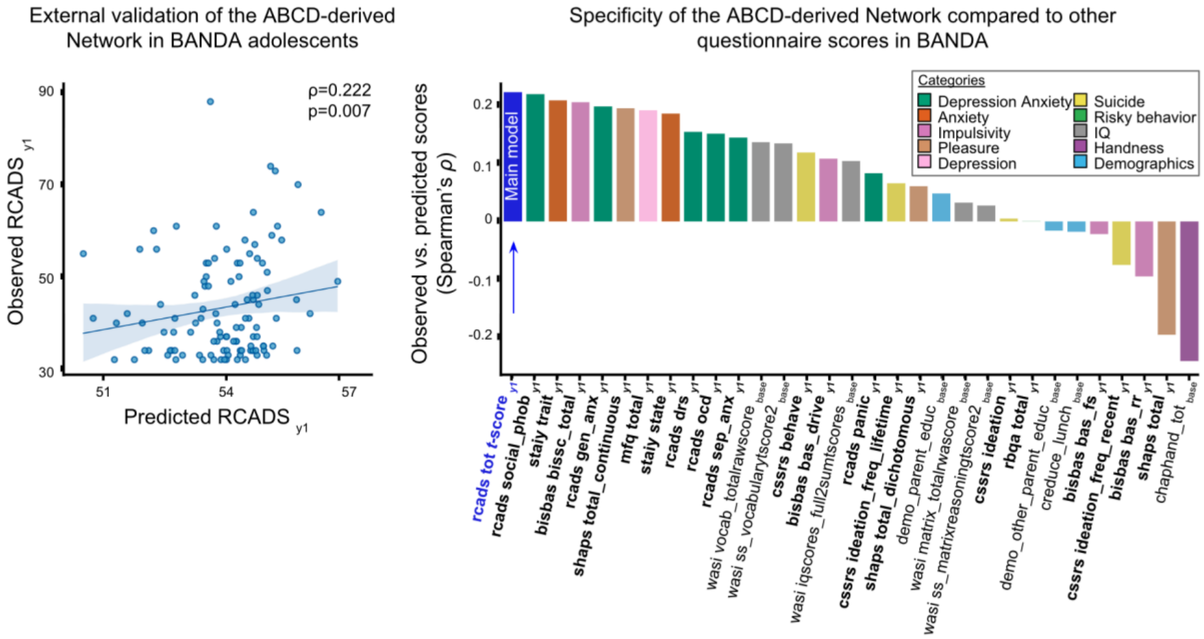

(**Left**) Observed vs. predicted RCADS$_{y1}$ scores in BANDA adolescents (n = 150). Predicted RCADS$_{y1}$ scores were generated from functional connectivity based on the ABCD-derived Symptoms Network. The generalizability of the Symptoms Network from ABCD to BANDA was assessed using Spearman's rank correlation to minimize the impact of symptom scores skewness (Spearman's $r = 0.222$, $p = 0.007$). The fitted trend line and 95% CI are shown for visualization purposes (Pearson's $r = 0.210$, $p = 0.011$). (**Right**) To assess the specificity of the Symptoms Network to internalizing symptoms (rather than to a more general vulnerability to psychopathology), the Symptoms Network was used to predict a range of self-reported measures in BANDA at the 1-year follow-up, including internalizing psychopathology, other psychopathology, cognitive measures, and general demographic measures. The barplot displays the Spearman's ρ correlation coefficients (y-axis) of the main model presented in the manuscript (i.e., RCADS$_{y1}$; blue bar, the first bar on the left) as it compares to all other measures (x-axis) acquired at the 1-year follow-up assessment and a few demographic measures acquired at baseline. Every model was corrected for RCADS$_{base}$, sex at birth, age, and mean head motion. Measures included the BIS-BAS (Carver & White, 1994) drive, reward responsiveness, and fun seeking subscales; Chapman Handedness Inventory (Chapman & Chapman, 1987), CSSRS (Posner et al., 2011), MFQ (Angold et al., 1995), RBQA (Auerbach & Gardiner, 2012); SHAPS (Carver & White, 1994), STAI (Spielberger et al., 1970), and WASI-II (Wechsler, 2018). BISBAS: Behavioral Inhibition and Behavioral Activation Questionnaire; CSSRS: Columbia Suicide Severity Rating Scale; y1: 1-year follow-up assessment; MFQ: Mood and Feelings Questionnaire; STAI: State-Trait Anxiety Inventory; RBQA: Risky Behavior Questionnaire for Adolescents; RCADS: Revised Child Anxiety and Depression Scale; SHAPS: Snaith-Hamilton Pleasure Scale; WASI-II: Wechsler Abbreviated Scale of Intelligence.

Figure S5. fMRI quality control distributions in BANDA.

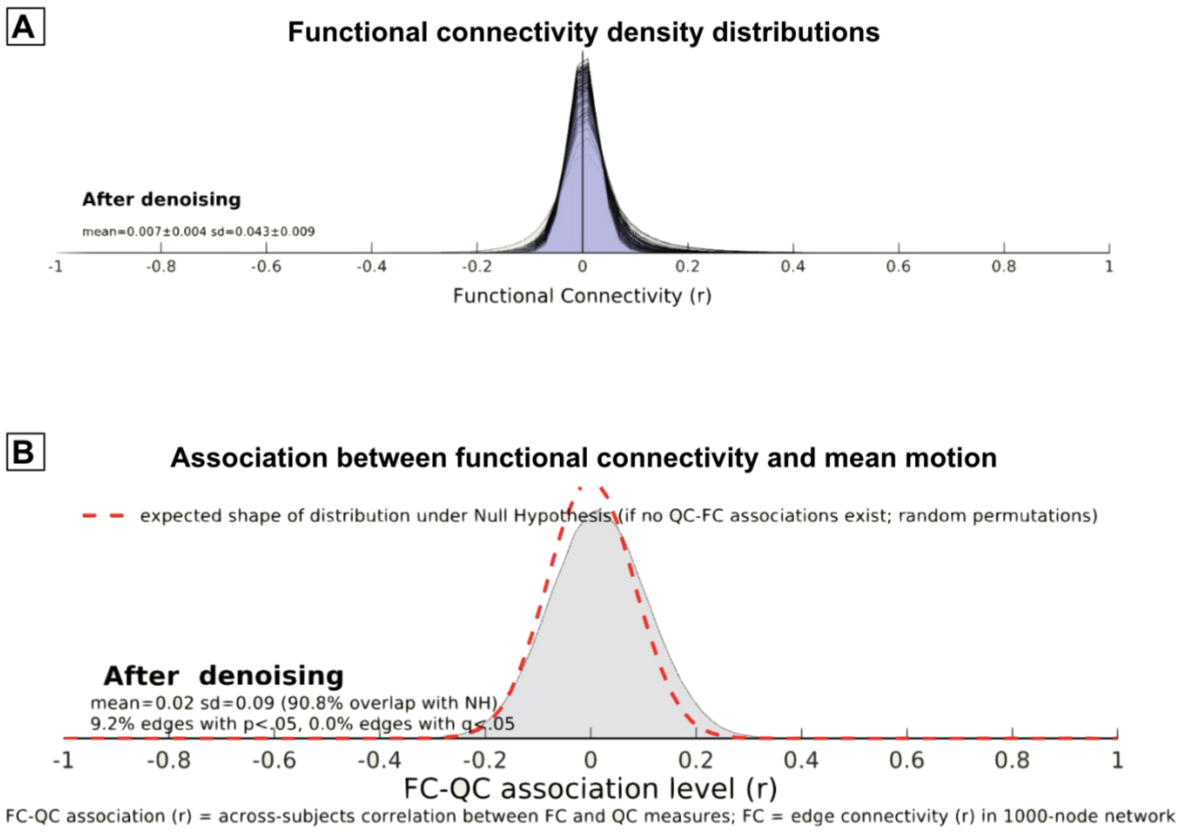

(**A**) Density distributions of functional connectivity strengths (*r* coefficients) after denoising between 1,000 randomly selected voxels for each participant (BANDA n = 150). (**B**) QC-FC correlation distribution between functional connectivity estimates and in-scanner mean head motion. The red dotted line represents a theoretical artifact-free null-hypothesis distribution (Morfini et al., 2023). Higher levels of overlap between the QC-FC distribution and the red dotted line (e.g., above 95%) can be considered indicative of negligible modulations in the connectivity correlation structure driven by a source of noise. FC: functional connectivity; NH: null hypothesis; QC: quality control.

Figure S6. Gordon parcellation assignment to Yeo 7 canonical networks.

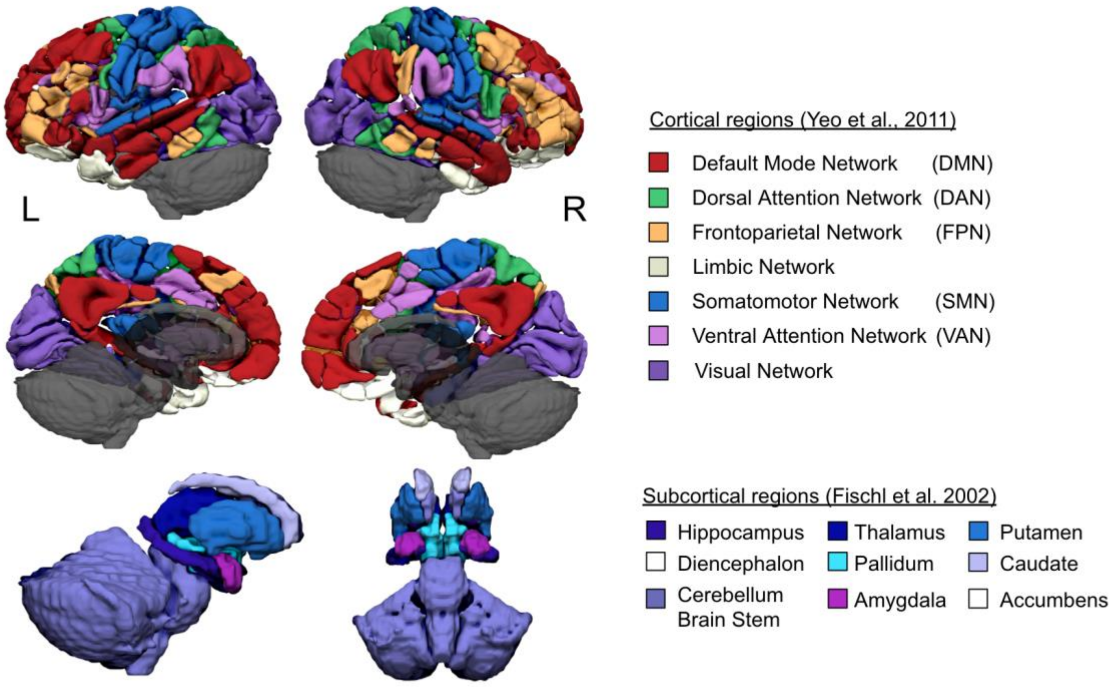

The brain images represent the 333 cortical Gordon parcels (Gordon et al., 2016) and the 19 subcortical parcels from the Freesurfer Atlas (Fischl et al., 2002) color-coded following the assignment to the canonical networks defined by Yeo and colleagues (Yeo et al., 2011).

Figure S7. Absolute network-network counts of the Symptoms Network.

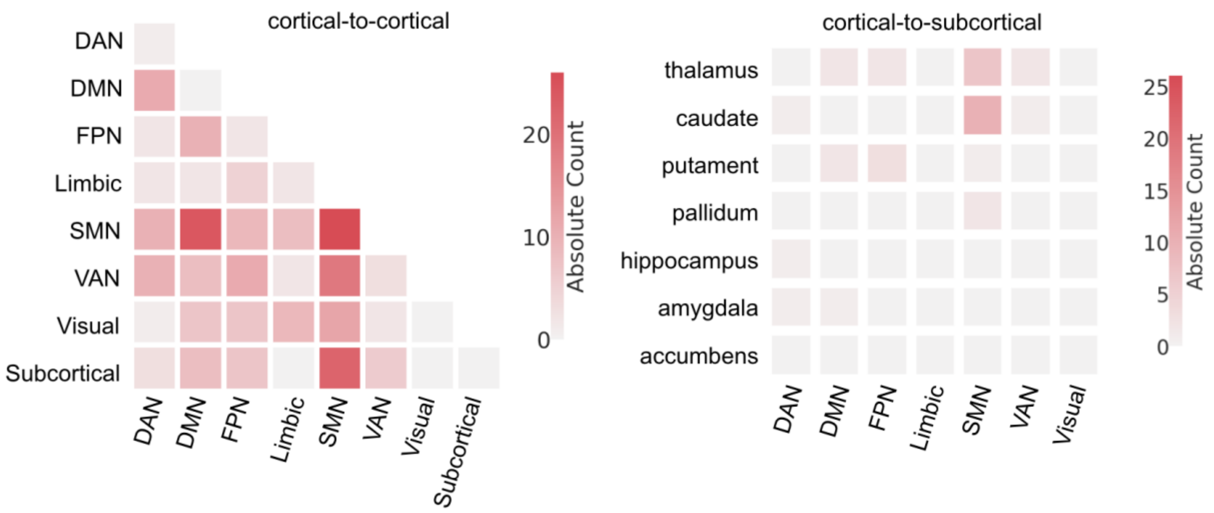

Absolute counts of the connections from the Symptoms Network. Counts are reported for cortical-to-cortical (**left**) and cortical-to-subcortical connections (**right**). DAN: Dorsal Attention Network; DMN: Default Mode Network; FPN: Frontoparietal Network; VAN: Ventral Attention Network; SMN: Somatomotor Network.

Figure S8. Within-participant between-connections mean functional connectivity of the subcortical-to-cortical connections of the Symptoms Network in BANDA.

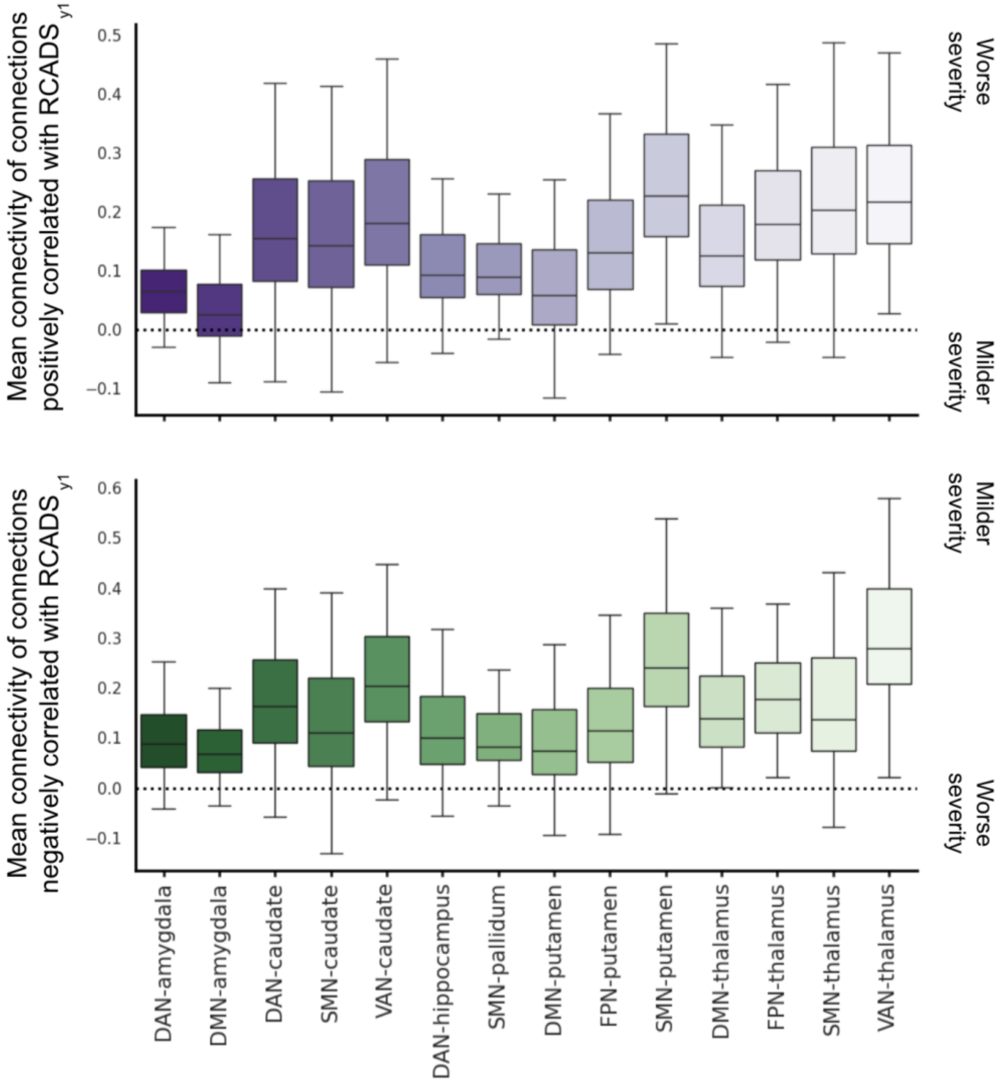

The plots represent the distributions of within-participant between-connection mean functional connectivity for every overrepresented subcortical-to-cortical-network pair of the Symptoms Network. For example, for every participant separately, we calculated the mean functional connectivity for all connections of a specific subcortical-to-cortical network pair. Each boxplot represents the distribution of the mean functional connectivity for a subset of connections for the included (n = 150) BANDA adolescents. To aid results interpretation, connections were split into a set of connections positively (purple, top) or negatively (green, bottom) correlated with $RCADS_{y1}$. For example, higher mean connectivity values in the boxplot for DAN-amygdala were correlated with worse $RCADS_{y1}$ severity in the positive connections (first purple boxplot) but were correlated with milder $RCADS_{y1}$ severity in the (first green boxplot). DAN: Dorsal Attention Network; DMN: Default Mode Network; FPN: Frontoparietal Network; SMN: Somatomotor Network; VAN: Ventral Attention Network.

Supplementary References

Achenbach, T. M. (1991). *Manual for the Child Behavior Checklist/4-18 and 1991 Profile*. Department of Psychiatry, University of Vermont.

American Psychiatric Association. (2013). *Diagnostic and statistical manual of mental disorders: DSM-5^{TM}, 5th ed* (pp. xliv, 947). American Psychiatric Publishing, Inc. https://doi.org/10.1176/appi.books.9780890425596

Angold, A., Costello, E. J., Messer, S. C., & Pickles, A. (1995). Development of a short questionnaire for use in epidemiological studies of depression in children and adolescents. *International Journal of Methods in Psychiatric Research*, *5*(4), 237–249.

Auerbach, R. P., & Gardiner, C. K. (2012). Moving beyond the trait conceptualization of self-esteem: The prospective effect of impulsiveness, coping, and risky behavior engagement. *Behaviour Research and Therapy*, *50*(10), 596–603. https://doi.org/10.1016/j.brat.2012.06.002

Barch, D. M., Albaugh, M. D., Avenevoli, S., Chang, L., Clark, D. B., Glantz, M. D., Hudziak, J. J., Jernigan, T. L., Tapert, S. F., Yurgelun-Todd, D., Alia-Klein, N., Potter, A. S., Paulus, M. P., Prouty, D., Zucker, R. A., & Sher, K. J. (2018). Demographic, physical and mental health assessments in the adolescent brain and cognitive development study: Rationale and description. *Developmental Cognitive Neuroscience*, *32*, 55–66. https://doi.org/10.1016/j.dcn.2017.10.010

Carver, C. S., & White, T. L. (1994). Behavioral inhibition, behavioral activation, and affective responses to impending reward and punishment: The BIS/BAS Scales. *Journal of Personality and Social Psychology*, *67*(2), 319–333. https://doi.org/10.1037/0022-3514.67.2.319

Casey, B. J., Cannonier, T., Conley, M. I., Cohen, A. O., Barch, D. M., Heitzeg, M. M., Soules, M. E., Teslovich, T., Dellarco, D. V., Garavan, H., Orr, C. A., Wager, T. D., Banich, M. T., Speer, N. K., Sutherland, M. T., Riedel, M. C., Dick, A. S., Bjork, J. M., Thomas, K. M., … Dale, A. M. (2018). The Adolescent Brain Cognitive Development (ABCD) study: Imaging acquisition across 21 sites. *Developmental Cognitive Neuroscience*, *32*, 43–54. https://doi.org/10.1016/j.dcn.2018.03.001

Chai, X. J., Castañón, A. N., Öngür, D., & Whitfield-Gabrieli, S. (2012). Anticorrelations in resting state networks without global signal regression. *NeuroImage*, *59*(2), 1420–1428. https://doi.org/10.1016/j.neuroimage.2011.08.048

Chapman, L. J., & Chapman, J. P. (1987). The measurement of handedness. *Brain and Cognition*, *6*(2), 175–183. https://doi.org/10.1016/0278-2626(87)90118-7

de Ross, R. L., Gullone, E., & Chorpita, B. F. (2002). The Revised Child Anxiety and Depression Scale: A Psychometric Investigation with Australian Youth. *Behaviour Change*, *19*(2), 90–101. https://doi.org/10.1375/bech.19.2.90

Feczko, E., Conan, G., Marek, S., Tervo-Clemmens, B., Cordova, M., Doyle, O., Earl, E., Perrone, A., Sturgeon, D., Klein, R., Harman, G., Kilamovich, D., Hermosillo, R., Miranda-Dominguez, O., Adebimpe, A., Bertolero, M., Cieslak, M., Covitz, S., Hendrickson, T., … Fair, D. A. (2021). *Adolescent Brain Cognitive Development (ABCD) Community MRI Collection and Utilities* (p. 2021.07.09.451638). bioRxiv. https://doi.org/10.1101/2021.07.09.451638

Fischl, B., Salat, D. H., Busa, E., Albert, M., Dieterich, M., Haselgrove, C., van der Kouwe, A., Killiany, R., Kennedy, D., Klaveness, S., Montillo, A., Makris, N., Rosen, B., & Dale, A. M. (2002). Whole Brain Segmentation: Automated Labeling of Neuroanatomical

Structures in the Human Brain. *Neuron*, *33*(3), 341–355. https://doi.org/10.1016/S0896-6273(02)00569-X

Garavan, H., Bartsch, H., Conway, K., Decastro, A., Goldstein, R. Z., Heeringa, S., Jernigan, T., Potter, A., Thompson, W., & Zahs, D. (2018). Recruiting the ABCD sample: Design considerations and procedures. *Developmental Cognitive Neuroscience*, *32*, 16–22. https://doi.org/10.1016/j.dcn.2018.04.004

Glasser, M. F., Sotiropoulos, S. N., Wilson, J. A., Coalson, T. S., Fischl, B., Andersson, J. L., Xu, J., Jbabdi, S., Webster, M., Polimeni, J. R., Van Essen, D. C., Jenkinson, M., & WU-Minn HCP Consortium. (2013). The minimal preprocessing pipelines for the Human Connectome Project. *NeuroImage*, *80*, 105–124. https://doi.org/10.1016/j.neuroimage.2013.04.127

Gordon, E. M., Laumann, T. O., Adeyemo, B., Huckins, J. F., Kelley, W. M., & Petersen, S. E. (2016). Generation and Evaluation of a Cortical Area Parcellation from Resting-State Correlations. *Cerebral Cortex*, *26*(1), 288–303. https://doi.org/10.1093/cercor/bhu239

Greene, A. S., Gao, S., Scheinost, D., & Constable, R. T. (2018). Task-induced brain state manipulation improves prediction of individual traits. *Nature Communications*, *9*(1), Article 1. https://doi.org/10.1038/s41467-018-04920-3

Hubbard, N. A., Bauer, C. C. C., Siless, V., Auerbach, R. P., Elam, J. S., Frosch, I. R., Henin, A., Hofmann, S. G., Hodge, M. R., Jones, R., Lenzini, P., Lo, N., Park, A. T., Pizzagalli, D. A., Vaz-DeSouza, F., Gabrieli, J. D. E., Whitfield-Gabrieli, S., Yendiki, A., & Ghosh, S. S. (2024). The Human Connectome Project of adolescent anxiety and depression dataset. *Scientific Data*, *11*(1), 837. https://doi.org/10.1038/s41597-024-03629-x

Hubbard, N. A., Siless, V., Frosch, I. R., Goncalves, M., Lo, N., Wang, J., Bauer, C. C. C., Conroy, K., Cosby, E., Hay, A., Jones, R., Pinaire, M., Vaz De Souza, F., Vergara, G., Ghosh, S., Henin, A., Hirshfeld-Becker, D. R., Hofmann, S. G., Rosso, I. M., … Whitfield-Gabrieli, S. (2020). Brain function and clinical characterization in the Boston adolescent neuroimaging of depression and anxiety study. *NeuroImage: Clinical*, *27*, 102240. https://doi.org/10.1016/j.nicl.2020.102240

Kaufman, J., Birmaher, B., Brent, D., Rao, U., Flynn, C., Moreci, P., Williamson, D., & Ryan, N. (1997). Schedule for Affective Disorders and Schizophrenia for School-Age Children-Present and Lifetime Version (K-SADS-PL): Initial Reliability and Validity Data. *Journal of the American Academy of Child & Adolescent Psychiatry*, *36*(7), 980–988. https://doi.org/10.1097/00004583-199707000-00021

Morfini, F., Whitfield-Gabrieli, S., & Nieto-Castañón, A. (2023). Functional connectivity MRI quality control procedures in CONN. *Frontiers in Neuroscience*, *17*. https://www.frontiersin.org/articles/10.3389/fnins.2023.1092125

Posner, K., Brown, G. K., Stanley, B., Brent, D. A., Yershova, K. V., Oquendo, M. A., Currier, G. W., Melvin, G. A., Greenhill, L., Shen, S., & Mann, J. J. (2011). The Columbia–Suicide Severity Rating Scale: Initial Validity and Internal Consistency Findings From Three Multisite Studies With Adolescents and Adults. *American Journal of Psychiatry*, *168*(12), 1266–1277. https://doi.org/10.1176/appi.ajp.2011.10111704

Shen, X., Finn, E. S., Scheinost, D., Rosenberg, M. D., Chun, M. M., Papademetris, X., & Constable, R. T. (2017). Using connectome-based predictive modeling to predict individual behavior from brain connectivity. *Nature Protocols*, *12*(3), Article 3. https://doi.org/10.1038/nprot.2016.178

Siless, V., Hubbard, N. A., Jones, R., Wang, J., Lo, N., Bauer, C. C. C., Goncalves, M., Frosch,

I., Norton, D., Vergara, G., Conroy, K., De Souza, F. V., Rosso, I. M., Wickham, A. H., Cosby, E. A., Pinaire, M., Hirshfeld-Becker, D., Pizzagalli, D. A., Henin, A., … Yendiki, A. (2020). Image acquisition and quality assurance in the Boston Adolescent Neuroimaging of Depression and Anxiety study. *NeuroImage: Clinical*, *26*, 102242. https://doi.org/10.1016/j.nicl.2020.102242

Smith, S. M., Beckmann, C. F., Andersson, J., Auerbach, E. J., Bijsterbosch, J., Douaud, G., Duff, E., Feinberg, D. A., Griffanti, L., Harms, M. P., Kelly, M., Laumann, T., Miller, K. L., Moeller, S., Petersen, S., Power, J., Salimi-Khorshidi, G., Snyder, A. Z., Vu, A. T., … Glasser, M. F. (2013). Resting-state fMRI in the Human Connectome Project. *NeuroImage*, *80*, 144–168. https://doi.org/10.1016/j.neuroimage.2013.05.039

Spielberger, C., Gorsuch, R., & Luschene, E. (1970). *Manual For the State-Trait Anxiety Interview (Self-Evaluation Questionnaire)*. https://scholar.google.com/scholar_lookup?title=Manual%20For%20the%20State-Trait%20Anxiety%20Interview%20&publication_year=1970&author=C.D.%20Spielberger&author=R.L.%20Gorsuch&author=E.%20Luschene

Tozzi, L., Staveland, B., Holt-Gosselin, B., Chesnut, M., Chang, S. E., Choi, D., Shiner, M., Wu, H., Lerma-Usabiaga, G., Sporns, O., Barch, D. M., Gotlib, I. H., Hastie, T. J., Kerr, A. B., Poldrack, R. A., Wandell, B. A., Wintermark, M., & Williams, L. M. (2020). The human connectome project for disordered emotional states: Protocol and rationale for a research domain criteria study of brain connectivity in young adult anxiety and depression. *NeuroImage*, *214*, 116715. https://doi.org/10.1016/j.neuroimage.2020.116715

Wechsler, D. (2018). *Wechsler Abbreviated Scale of Intelligence—Second Edition* [Dataset]. https://doi.org/10.1037/t15171-000

Whitfield-Gabrieli, S., & Nieto-Castanon, A. (2012). *Conn: A Functional Connectivity Toolbox for Correlated and Anticorrelated Brain Networks | Brain Connectivity*. https://www.liebertpub.com/doi/abs/10.1089/brain.2012.0073

Yeo, T. B. T., Krienen, F. M., Sepulcre, J., Sabuncu, M. R., Lashkari, D., Hollinshead, M., Roffman, J. L., Smoller, J. W., Zöllei, L., Polimeni, J. R., Fischl, B., Liu, H., & Buckner, R. L. (2011). The organization of the human cerebral cortex estimated by intrinsic functional connectivity. *Journal of Neurophysiology*, *106*(3), 1125–1165. https://doi.org/10.1152/jn.00338.2011
